# Targeted long-read sequencing to quantify methylation of the *C9orf72* repeat expansion

**DOI:** 10.1186/s13024-024-00790-0

**Published:** 2024-12-21

**Authors:** Evan Udine, NiCole A. Finch, Mariely DeJesus-Hernandez, Jazmyne L. Jackson, Matthew C. Baker, Siva Arumugam Saravanaperumal, Eric Wieben, Mark T.W. Ebbert, Jaimin Shah, Leonard Petrucelli, Rosa Rademakers, Björn Oskarsson, Marka van Blitterswijk

**Affiliations:** 1 Department of Neuroscience, Mayo Clinic, Jacksonville, FL USA; 2 Mayo Clinic Graduate School of Biomedical Sciences, Mayo Clinic, Jacksonville, FL USA; 3 Fels Cancer Institute for Personalized Medicine, Temple University, Lewis Katz School of Medicine, Philadelphia, PA USA; 4 Genome Analysis Core, Mayo Clinic, Rochester, MN USA; 5 Department of Neuroscience, University of Kentucky Sanders-Brown Center on Aging, Lexington, KY USA; 6 Department of Neurology, Mayo Clinic, Jacksonville, FL USA; 7 VIB Center for Molecular Neurology, Antwerp, Belgium; 8 Department of Biomedical Science, University of Antwerp, Antwerp, Belgium

**Keywords:** C9orf72, Long-read sequencing, Methylation, Repeat expansions, Amyotrophic lateral sclerosis, Frontotemporal dementia

## Abstract

**Background:**

The gene *C9orf72* harbors a non-coding hexanucleotide repeat expansion known to cause amyotrophic lateral sclerosis and frontotemporal dementia. While previous studies have estimated the length of this repeat expansion in multiple tissues, technological limitations have impeded researchers from exploring additional features, such as methylation levels.

**Methods:**

We aimed to characterize *C9orf72* repeat expansions using a targeted, amplification-free long-read sequencing method. Our primary goal was to determine the presence and subsequent quantification of observed methylation in the *C9orf72* repeat expansion. In addition, we measured the repeat length and purity of the expansion. To do this, we sequenced DNA extracted from blood for 27 individuals with an expanded *C9orf72* repeat.

**Results:**

For these individuals, we obtained a total of 7,765 on-target reads, including 1,612 fully covering the expanded allele. Our in-depth analysis revealed that the expansion itself is methylated, with great variability in total methylation levels observed, as represented by the proportion of methylated CpGs (13 to 66%). Interestingly, we demonstrated that the expanded allele is more highly methylated than the wild-type allele (P-Value = 2.76E-05) and that increased methylation levels are observed in longer repeat expansions (P-Value = 1.18E-04). Furthermore, methylation levels correlate with age at collection (P-Value = 3.25E-04) as well as age at disease onset (P-Value = 0.020). Additionally, we detected repeat lengths up to 4,088 repeats (~ 25 kb) and found that the expansion contains few interruptions in the blood.

**Conclusions:**

Taken together, our study demonstrates robust ability to quantify methylation of the expanded *C9orf72* repeat, capturing differences between individuals harboring this expansion and revealing clinical associations.

**Supplementary Information:**

The online version contains supplementary material available at 10.1186/s13024-024-00790-0.

## Background

The most frequently observed genetic cause of amyotrophic lateral sclerosis (ALS) and frontotemporal dementia (FTD) is a non-coding hexanucleotide (GGGGCC) repeat expansion in the gene *C9orf72* [1, 2]. Typically, having less than 30 repeats is not considered to be pathogenic, and most often patients carry hundreds to thousands of repeats [1–3]. Studies have estimated that this repeat expansion is present in 40% of familial and 5–7% of sporadic ALS patients [4], as well as 20–25% of familial and 5–10% of sporadic FTD cases [5], though this differs by population [6]. Thus far, three primary pathogenic mechanisms have been identified, including both loss- and gain-of-function hypotheses. In terms of loss-of-function, we and others have shown that *C9orf72* gene expression is reduced in multiple brain regions of patients with the repeat expansion [1, 7–12]. Proposed gain-of-function mechanisms include the presence of nuclear RNA foci composed of the repetitive RNA, which sequester and thus disrupt the activity of RNA-binding proteins [13–17] and the production of dipeptide repeat proteins (DPRs), which are translated from the expansion itself via repeat associated non-AUG (RAN) translation and have the potential to be neurotoxic [18–22].

Currently, the repeat expansion is typically identified using PCR-based assays; however, Southern blotting can be used to not only detect, but also estimate the length of the expansion [1, 23, 24]. These methods do not allow for researchers to quantify other features of the expansion, such as DNA methylation or the actual sequence content. Attempts to use next-generation sequencing methods to understand the repeat expansion at a greater depth have had varying degrees of success. Short-read sequencing can detect the repeat expansion but is unable to reliably reassemble the full expansion due to the length and high GC content [25]. Long-read sequencing approaches have shown promise in identifying and capturing the full length of repeat expansions [26–35]. The two predominant long-read sequencing technologies have been developed by Pacific Biosciences (PacBio) and Oxford Nanopore Technologies (ONT). PacBio utilizes single-molecule real-time (SMRT) sequencing to generate extremely accurate reads by annealing circular primers to the end of each DNA molecule. This allows each molecule of DNA to be sequenced multiple times by the same polymerase, maximizing accuracy [36, 37]. ONT, on the other hand, has developed a nanopore-based sequencing method that relies on a motor protein pulling a single molecule of DNA through a pore. At the pore, the ionic current is measured, which differs with each unique base [38]. Interestingly, neither of these approaches require amplified DNA and both platforms have developed methods for targeted sequencing using CRISPR Cas9. This is important because long, GC-rich repeat expansions, like that in *C9orf72*, cannot be amplified. Several studies have successfully used long-read sequencing to study *C9orf72* [28, 31, 39, 40]. One previous study compared PacBio and ONT whole-genome sequencing approaches, as well as PacBio’s targeted no-amplification (No-Amp) sequencing method [31]. There, PacBio targeted long-read sequencing seemed to provide the most reliable and highest coverage for the *C9orf72* repeat expansion, especially in human subjects. In addition, further studies have demonstrated that this method provides accurate length estimates in both human cerebellar tissue [28] and induced pluripotent stem cells (iPSCs) [39], when compared to Southern blot estimates. Furthermore, studies of additional repeat expansion disorders have utilized PacBio targeted, long-read sequencing to accurately identify, size, and characterize variability of repeat expansions [27, 29, 30].

One of the primary benefits of long-read sequencing is its ability to accurately provide estimates of base modifications [41, 42]. Specifically, PacBio’s No-Amp sequencing currently allows researchers to quantify 5-methylcytosine (5mC) by measuring the kinetics of the polymerase that incorporates labelled nucleotides for sequencing on the instrument. Importantly, previous studies have demonstrated that 5mC calling is highly reliable when comparing sequencing methods. For example, strong correlations have been observed between PacBio and bisulfite sequencing (~ 0.90), as well as between PacBio and ONT (~ 0.89) [41, 42]. The few studies that have utilized this technology have not evaluated these levels for *C9orf72*. Multiple studies have, however, used other methods to evaluate *C9orf72* promoter methylation. There, it has been observed that hypermethylation of the promoter in individuals with the expansion is associated with a decrease in transcript levels and corresponding pathologies [8, 43–46]. Evidence of methylation of the *C9orf72* repeat expansion itself has also been demonstrated using a PCR-based method [47], though the amount was not quantified. Notably, GC-rich expansions are known to be methylated, such as *FMR1* (CGG), where in the full mutation (> 200 repeats), methylation is seen not only at an upstream CpG island, but of the repeat itself, leading to loss of expression of the protein [48–50]. Furthermore, a similar phenomenon has been observed in other GC-rich triplet repeats, such as those in *FRAXE*, *FRA2A*, *FRA7A* and *FRA12A* [51–53]. Therefore, one wonders whether and to what extent the *C9orf72* repeat expansion is methylated and how this may affect clinical progression and known pathologies.

Another advantage of long-read sequencing is its ability to provide accurate measurements of expansion length and the sequence content. Previous studies have shown that the length of this repeat expansion is associated with various clinico-pathological characteristics, including age at onset, survival time, and DPR burden [23, 28]. Unlike many repeat expansions, anticipation is not commonly observed, with familial studies of repeat length even identifying frequent paternally inherited contractions in blood [8, 54]. However, the expansion length in blood remains difficult to size as it generally appears as a smear on Southern blot, has been correlated with age at collection, and may change over time [8, 23, 54–56]. An additional advantage of long-read sequencing is that it allows us to examine the full-length sequence to identify interruptions, which are known to act as disease modifiers in other neurological diseases [57, 58].

Here, we aim to use targeted long-read sequencing to comprehensively characterize the *C9orf72* expansion in blood, evaluating the methylation levels, repeat length, and sequence content of the *C9orf72* repeat expansion.

## Methods

### Participants - biological specimens

Blood specimens were obtained from the ALS Center at Mayo Clinic in Florida. Our cohort included 34 samples from 27 unique individuals with the *C9orf72* repeat expansion. We included 15 ALS patients (53% female, 63 years old [median]), 1 FTD patient (0% female, 73 years old), and 11 pre-symptomatic individuals (73% female, 39 years old). Longitudinal specimens were available for 6 individuals corresponding to 13 different time points. Individuals were selected based on our previous Southern blotting study, which also measured *C9orf72* promoter methylation levels [8]. See Table 1 for more information. In addition, we leveraged previously published data that was generated from cerebellar brain tissue for 28 subjects [28]. Further information about that cohort can be found elsewhere [28].

**Table 1:** Cohort overview for individuals included in the primary analysis of blood long-read sequencing data. We included samples from a total of 27 individuals, including 16 symptomatic individuals and 11 pre-symptomatic individuals. Out of 27 individuals, 16 were female. Percentages are shown in parentheses for diagnoses and sex. The median values are shown for age at collection, age at onset, repeat length, and promoter methylation and the interquartile range (IQR) is displayed in parentheses. We obtained longitudinal specimens from 6 individuals, corresponding to 13 unique time points

Variable	Subjects (*n* = 27)
ALS, *n* (%)	15 (55.56)
FTD, *n* (%)	1 (3.70)
Pre-symptomatic, *n* (%)	11 (40.74)
Sex, *n* (% female)	16 (59.26)
Age at collection, *median* (IQR)	57.19 (43.33–64.11)
Age at onset (ALS), *median* (IQR)	61.42 (57.17–65.39)
Repeat length, *median* (IQR)	18.53 (8.31–23.32)
Promoter methylation, *median* (IQR)	1.93 (0.90–11.92)

### Long-read sequencing

**Fig. 1:**
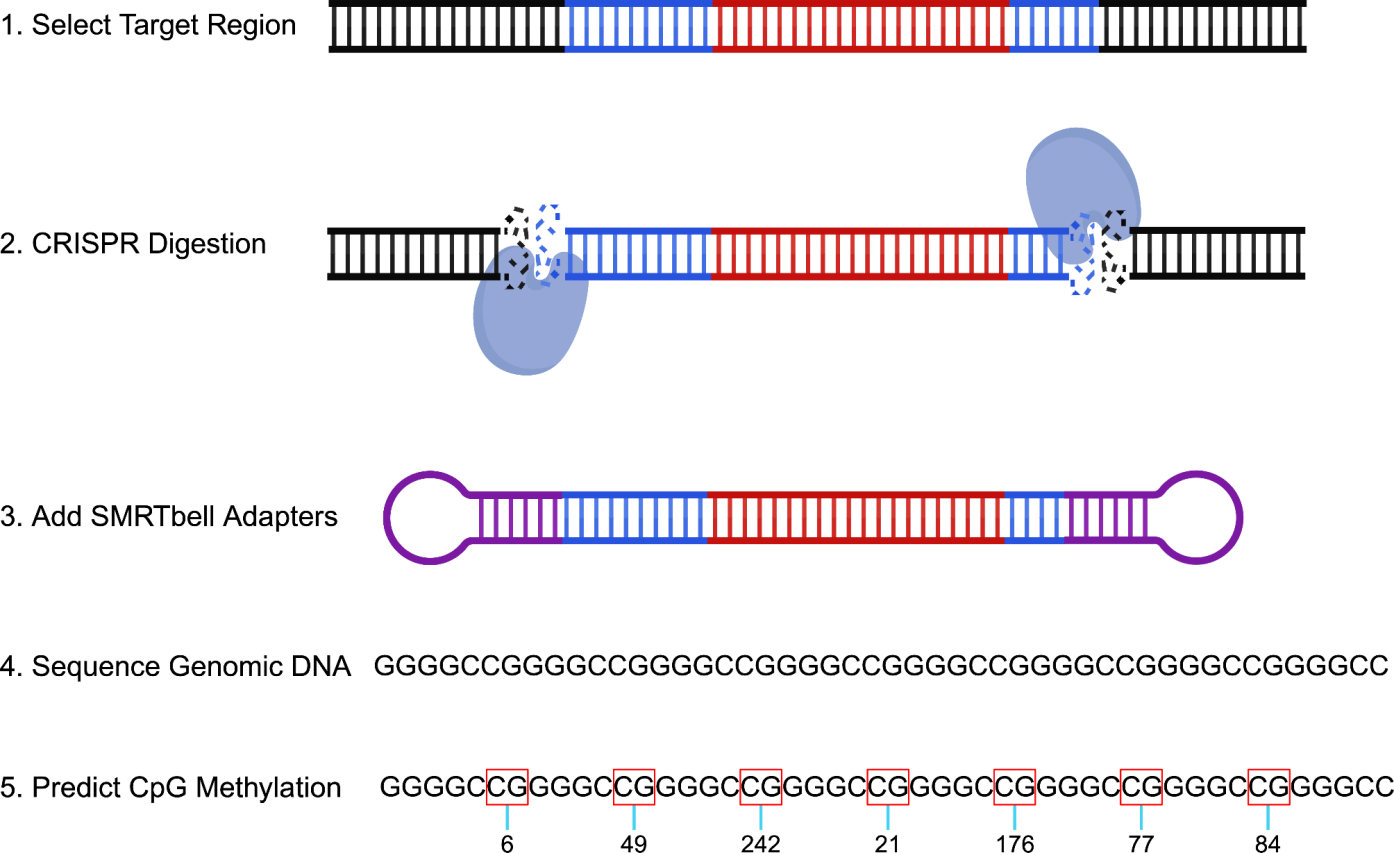
Schematic overview of No-Amp sequencing with emphasis on calculating methylation. The DNA colored blue denotes the flanking region surrounding the *C9orf72* repeat. The DNA colored red represents the *C9orf72* repeat itself. The purple circular adapters exemplify SMRTbell Adapters. Numbers below the boxed CGs represent methylation probabilities. The target region was obtained following the No-Amp targeted sequencing method (PN 101-801-500 Version 09, Jan 2022). This figure was created using https://BioRender.com

We completed targeted long-read sequencing of genomic DNA for a region that includes the *C9orf72* repeat expansion as previously described (Fig. 1) [28, 31]. For the blood specimens, high-molecular-weight genomic DNA was extracted from frozen blood after adding RBC lysis buffer (Puregene), using the Nanobind CBB kit (SKU 102-301-900; PacBio). DNA QC was performed using Nanodrop Absorbance (Thermo Scientific) and double-stranded DNA concentrations were measured using a Qubit 2.0 Fluorometer (Invitrogen). Genomic DNA (up to 10 µg) was enriched for *C9orf72* repeat-containing SMRTbell™ templates using PacBio’s No-Amp targeted sequencing method (PN 101-801-500 Version 09, Jan 2022) at the Mayo Clinic Genome Analysis Core. DNA was treated with recombinant shrimp alkaline phosphatase (M0371S, New England Biolabs) to exclude fragment ends from downstream ligation steps prior to SMRTbell library preparation. We used both sense and antisense CRISPR RNA plus trans-activating CRISPR RNA along with the Cas9 enzyme (Integrated DNA Technologies) for the digestion to excise the area of interest at 37 °C for 1 h. The digested DNA was ligated with blunt adapters and T4 DNA ligase at 16 °C for 2 h to produce SMRTbell templates. Partially and non-ligated products were reduced using a five-enzyme exonuclease digestion at 37 °C for 2 h. Exonuclease enzymes were removed by a trypsin (EMS0004, Sigma-Aldrich) treatment at 37 °C for 20 min, and followed by two AMPure^®^ PB bead purifications (PacBio). Primer v4 (PacBio) was then annealed to the library at 20 °C for 1 h and the Sequel^®^ II DNA polymerase 2.2 (PacBio) was bound to the library at 30 °C for 4 h. SMRTbell libraries were subsequently sequenced on a PacBio Sequel^®^ II with Sequel^®^ II 2.0 chemistry. One SMRT Cell (8 M) was used for each sample, with a 0.5-h extension, 4-h immobilization, and 30-h movie time.

### Long-read sequencing analysis

Sequencing data obtained from the blood were primarily analyzed using PacBio’s RepeatAnalysisTools pipeline, which was obtained via GitHub and organized into a conda environment. First, circular consensus sequencing (ccs) reads were generated from subreads using the pbccs package (v6.0.0). For all analyses, we used a signal to noise ratio threshold of 2.5, a minimum read length of 10 bp, and a maximum read length of 100 kb. We generated ccs reads separately for methylation/repeat purity and repeat length analyses. Consistent with our previous targeted long-read sequencing study [28], to capture repeat methylation and purity, we included ccs reads that had a minimum of 7 full passes and predicted accuracy of 99% or more. To measure repeat length, we included ccs reads that had at least 1 full pass and a predicted accuracy of 80% or more. Reads were then aligned to human reference genome GRCh38 using pbmm2 (v1.4.0). We determined the number of on-target zero-mode waveguides and generated coverage plots to visualize on-target reads. Two clusters were identified using K-means clustering of sequence kmer counts in the region of interest (chr9:27,573,437–27,573,598) and were split by allele (wild-type and expanded allele). Reads were required to include the flanking region on each side of the expansion. Because any truncated reads were removed, only reads containing the full expansion were included in downstream analyses. Methylation analyses were completed after generating ccs reads with hifi-kinetics. Then, 5mC sites were called using pbjasmine (v2.0.0) and per site methylation probabilities were estimated using pb-CpG-tools (v2.3.1). Following methylation calling, reads were processed as described above. A custom R script (v4.3.0) was used for further analysis and visualization of methylation data. The methylation probability was presented as a score ranging from 0 to 255, where higher scores corresponded to increased methylation probability at each CpG (Fig. 1). We summarized methylation data in 2 ways. First, we calculated the median methylation score per read and subsequently determined the median of all reads for each sample. Second, we calculated the proportion of methylated CpG sites to total number of CpGs, considering CpGs with a methylation score of **≥** 128 - top 50% - to be likely methylated. Similarly, we calculated the median proportion of methylated CpGs per read and subsequently determined the median of all reads for each sample. Every read was visualized in a waterfall-like plot including all CpG positions. Additionally, for our length analysis, each read was visualized for every sample by generating a waterfall plot colored by repeat motif. Our previously described custom python script [28] was then used for further analysis of expansion length and purity. See data and code availability for more information.

### Statistical analyses and figures

Data was summarized per sample for methylation levels, length, and sequence purity (Tables S1-4). For our methylation and purity analyses, when multiple reads were available for a given sample, we focused on the median and also reported the range (minimum to maximum). For analysis of repeat length, the maximum was used, since that measurement appeared to be most consistent with our Southern blot estimates [8]. We calculated Spearman’s rank correlation coefficient, Wilcoxon rank-sum test, and/or linear regression when appropriate for the nature of a given test, as indicated in the results. All statistical tests were two-sided and performed using R Statistical Software (v4.3.0).

## Results

### Blood long-read sequencing overview

We completed amplification-free, targeted, long-read sequencing of the *C9orf72* repeat expansion on DNA extracted from blood for 27 individuals known to harbor the repeat expansion (Table 1). We captured both the wild-type and expanded allele for all subjects. Overall, using our less stringent filtering criteria, we captured 7,765 reads covering this region. Of those reads, 6,153 mapped to the wild-type allele, while 1,612 mapped to the expanded allele (Fig. S1a), and 20/27 (74%) individuals had at least 10 reads fully spanning the expansion (Fig. S1b).

### Methylation

#### Blood overview

**Fig. 2:**
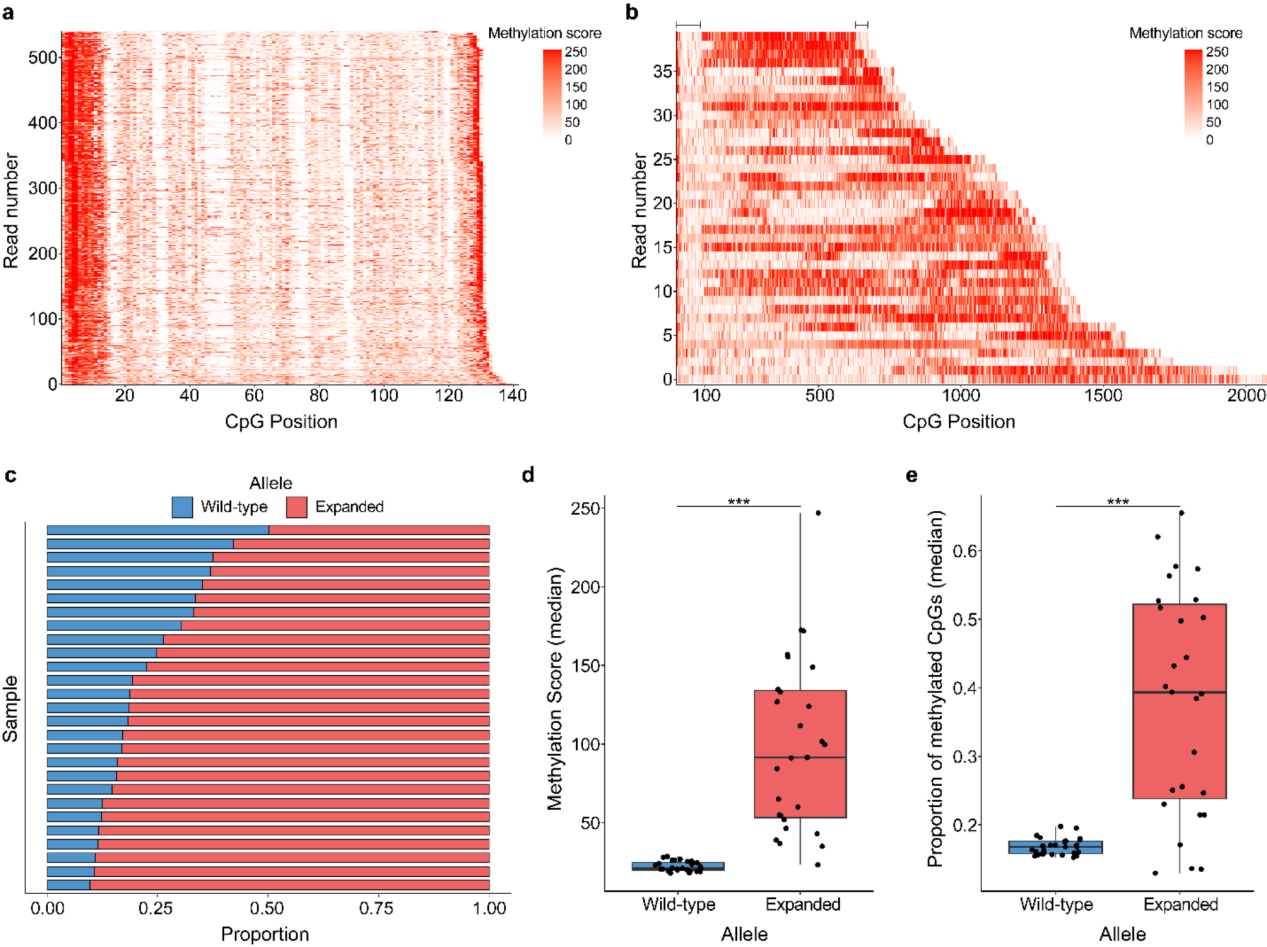
Methylation of the *C9orf72* repeat expansion. (**a-b**) Waterfall-like plots for (**a**) the wild-type and (**b**) expanded alleles (with flanking region) for one representative individual. The x-axis represents the position of each CpG within a read and the y-axis displays all reads sorted by number of CpG sites. Low methylation scores are presented in white and higher scores in red. Lines at the top of the waterfall-like plots indicate the approximate size of the flanking regions for the expanded allele. (**c**) Barplot showing the proportion of methylation (measured by median methylation score per read) per individual (*n* = 27) for the wild-type and expanded alleles. (**d-e**) Boxplot(s) displaying (**d**) the median methylation score and (**e**) median proportion of methylated CpGs per read for each individual (*n* = 27) for each allele. Boxes represent the interquartile range (IQR; 25th − 75th percentile), lines represent the median, and each dot corresponds to one individual. Significantly higher methylation was detected for the expanded allele using the methylation score (P-Value = 6.64E-06) and proportion of methylated CpGs (P-Value = 2.76E-05). A paired Wilcoxon rank-sum test was used for each of these comparisons. ***P-Value < 0.001

Our primary analysis was focused on measuring the methylation levels of the *C9orf72* repeat expansion and surrounding region. For the methylation analysis, we obtained a total of 4,313 reads mapping to the wild-type allele (Table S1) and 776 reads mapping to the expanded allele (Table S2). Of note, when calculating the proportion of methylated CpGs for these reads, we decided to define individual CpG sites with a methylation score > 50% as methylated. However, we would like to emphasize that a strong correlation was observed for each allele when using another threshold, the > 75% (wild-type: *r* = 0.59, P-Value = 0.001; expanded: *r* = 0.97, P-Value = 2.29E-16; Fig. S1c-d). We determined that both the wild-type allele (Fig. 2a, S2a-b) and expanded allele (Fig. 2b, S3a-b) contain CpG sites that are methylated and that notable variation in the amount of methylation of the expanded allele exists between individuals.

**Fig. 3:**
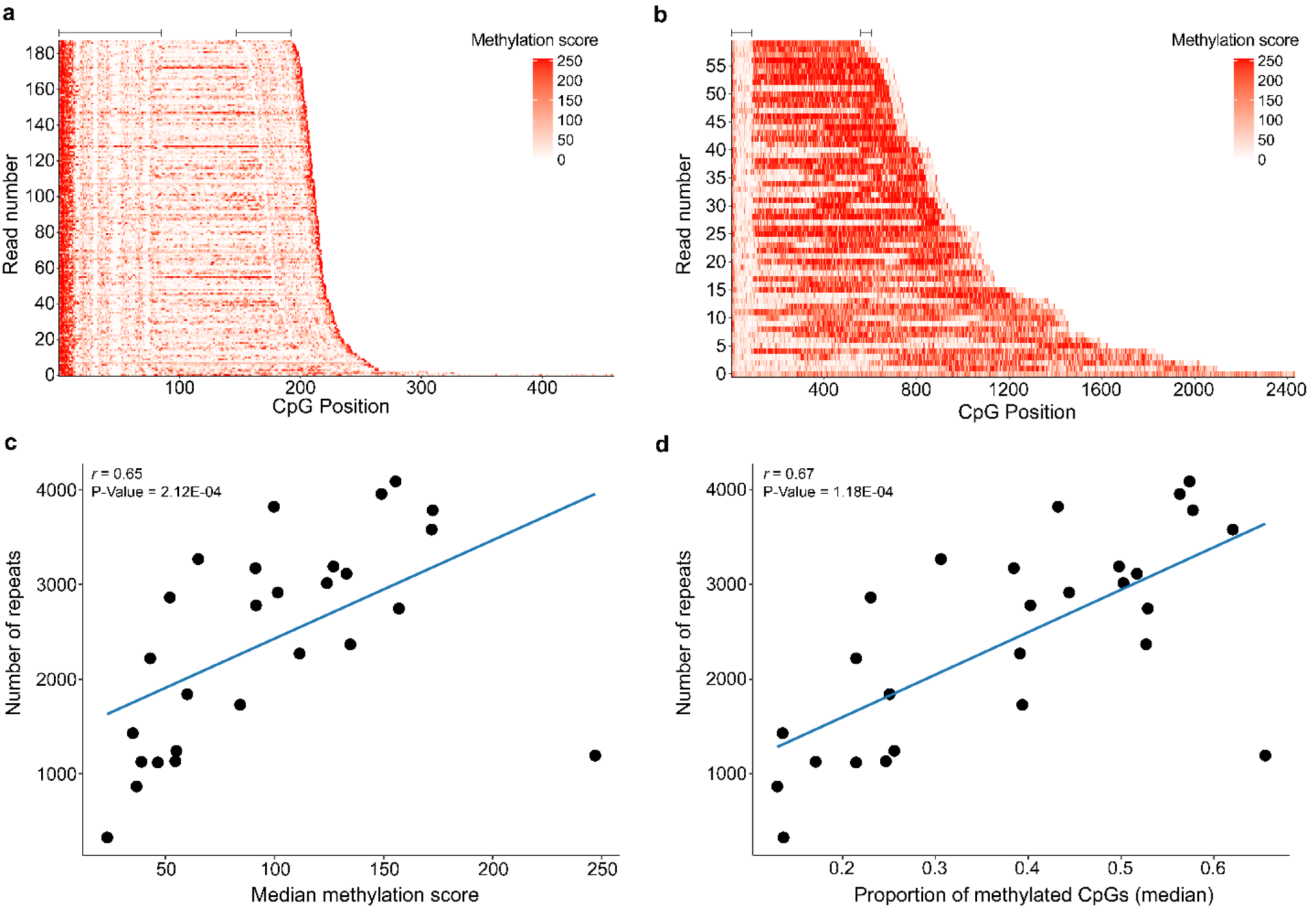
Methylation levels of various repeat sizes. (**a-b**) Waterfall-like plots for the expanded allele (with flanking region) for the samples with the (**a**) smallest and (**b**) longest repeat expansions in the cohort. The x-axis represents the position of each CpG within a read and the y-axis displays all reads sorted by number of CpG sites. Low methylation scores are presented in white and higher scores in red. Lines at the top of the waterfall-like plots indicate the approximate size of the flanking regions for the expanded allele. (**c-d**) Scatterplots showing the correlation between the maximum repeat length as determined using long-read sequencing and the (**c**) median methylation score and (**d**) the median proportion of methylated CpGs per read for all individuals (*n* = 27). Each dot represents an individual. A significant positive correlation was detected with the median methylation score (*r* = 0.65, P-Value = 2.12E-04) and median proportion of methylated CpGs (*r* = 0.67, P-Value = 1.18E-04). The solid blue line represents a linear regression line. A Spearman’s rank correlation was used for these analyses

For the wild-type allele, the median methylation score at CpG sites was 21.0 (18.0 to 28.5; Fig. S2c, Table S1) and the median proportion of methylated CpGs was 17% (15 to 20%; Fig. S2d, Table S1). For the expanded allele, the median methylation score at CpGs was 91.5 (23.3 to 247; Fig. S3c, Table S2) and the median proportion of methylated CpGs was 39% (13 to 66%; Fig. S3d, Table S2). Comparing the wild-type allele to the expanded allele revealed that the expanded allele had significantly higher methylation levels based on methylation scores (P-Value = 6.64E-06; Fig. 2c-d) and the proportion of methylated CpGs (P-Value = 2.76E-05; Fig. 2e). For both alleles, there appeared to be highly methylated CpGs at the beginning and end of each read (Fig. 2a, S2a-b, S4a-d). For the wild-type allele, less methylation was observed throughout the rest of the reads (Fig. 2a, S2a-b), while in the expanded allele for most samples, methylated CpGs could be observed throughout the reads (Fig. 2b, S3a-b). Remarkably, the individual with the smallest expansion length in this cohort (median number of repeats = 84.5, range = 64 to 327) had the lowest methylation score for the expanded allele and one of the lowest proportions of methylated CpGs (Fig. 3a, S3c-d, S5a-c, Tables S1-2) across all samples. Visual comparison of the expanded allele in the individual with the smallest expansion length compared to the longest expansion length demonstrated a clear difference in methylation pattern (Fig. 3a-b). To further explore the relationship between repeat length and methylation level we completed a correlation between repeat length (*see **repeat length for further information*) and the median methylation score and median proportion of methylated CpGs. We detected a significant positive correlation for both methylation score (*r* = 0.65, P-Value = 2.12E-04; Fig. 3c) and the proportion of methylated CpGs (*r* = 0.67, P-Value = 1.18E-04; Fig. 3d); therefore, individuals with longer expansions tended to have more highly methylated expansions.

#### Blood associations

**Fig. 4:**
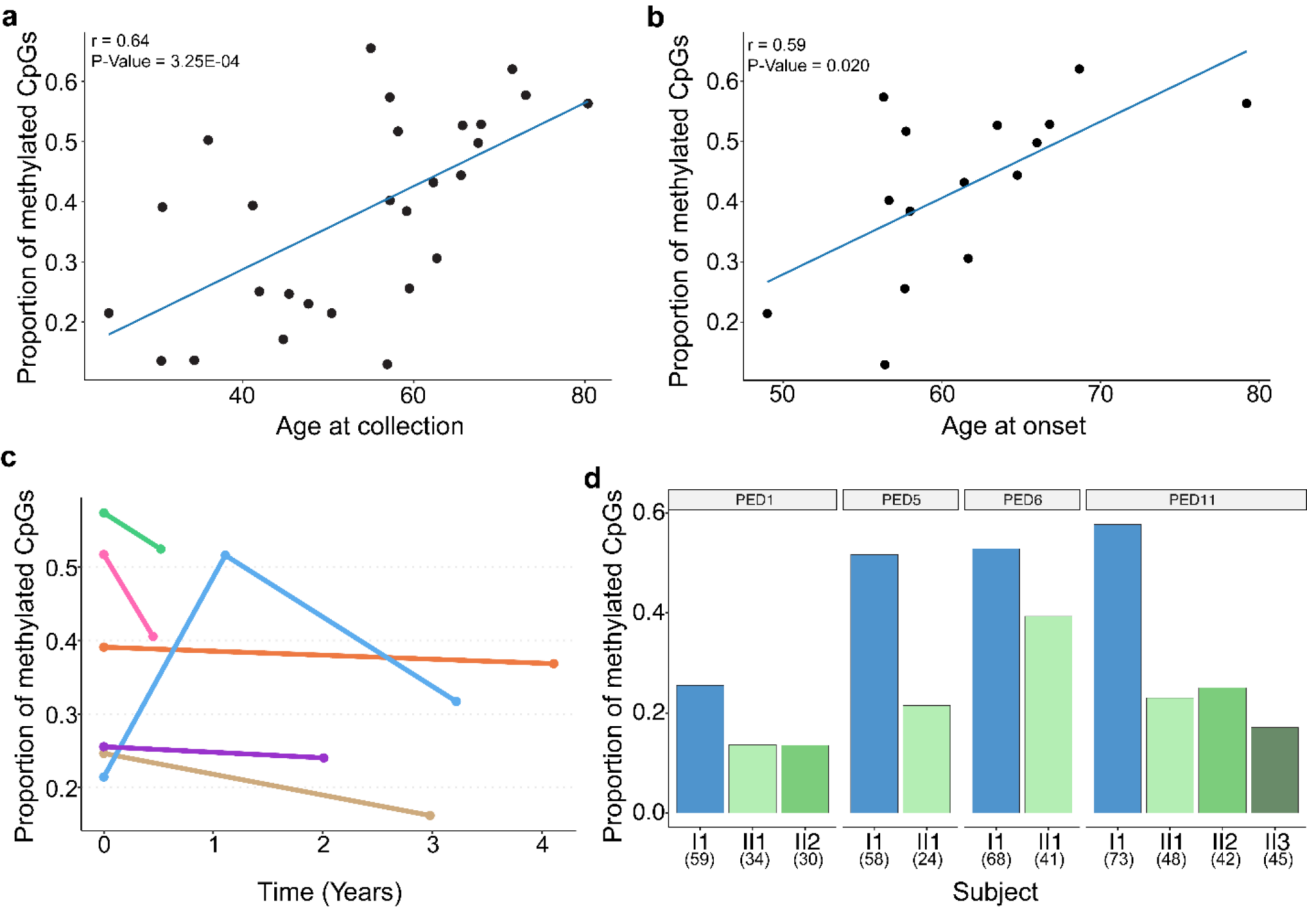
Methylation age-related, longitudinal and familial analyses. (**a**) Scatterplot showing the median proportion of methylated CpGs per read for each individual (*n* = 27) for the expanded allele and the age at collection. A significant positive correlation was detected (*r* = 0.64, P-Value = 3.25E-04). The solid blue line represents a linear regression line. Each dot represents one individual. (**b**) Scatterplot showing the median proportion of methylated CpGs per read for patients with ALS (*n* = 15) for the expanded allele and age at onset. A significant positive correlation was detected with age at onset (*r* = 0.59, P-Value = 0.020). The solid blue line represents a linear regression line. Each dot represents one individual. A Spearman’s rank correlation was used for these analyses. (**c**) Dotplot showing the median proportion of methylated CpGs per read for each individual for the expanded allele over time measured in years. Longitudinal measurements were obtained for 6 individuals. Each dot represents a unique time point and lines connect the points within a given individual. Each color corresponds to one individual. (**d**) Barplot(s) showing median proportion of methylated CpGs per read for each individual across 4 different pedigrees corresponding to 7 unique transmissions. Each pedigree was shown to display a paternally inherited contraction in our previous Southern blotting study. Paternal parents are presented as blue bars and offspring are presented in various shades of green. A decrease in the proportion of methylated CpGs was observed for all 7 transmissions. Pedigree numbers are presented above each barplot and match the pedigrees in our Southern blotting study. Numbers in parentheses represent age at collection for each individual

To evaluate the relevance of the variation we detected in methylation levels of the expanded allele, we examined the presence of associations with clinical variables. For these analyses, we focused on the proportion of methylated CpGs per subject. We determined that the proportion of methylated CpGs was significantly positively correlated with age at collection in our cohort of 27 subjects (*r* = 0.64, P-Value = 3.25E-04; Fig. 4a). In addition, the proportion of methylated CpGs appeared to be lower in pre-symptomatic individuals compared to symptomatic individuals (P-Value = 0.041; Fig. S6a). Since pre-symptomatic individuals tended to be younger than symptomatic individuals (P-Value = 1.97E-05; Fig. S6b), we then completed a multivariable linear regression analysis and found that when adjusting for age at collection, we did not detect a significant difference between the groups anymore (pre-symptomatic vs. symptomatic; P-Value = 0.24). Meanwhile, the association with age at collection remained significant (P-Value = 0.004). In the subset of patients affected by ALS (*n* = 15), we also noted a positive correlation between the proportion of methylated CpGs and age at collection (*r* = 0.62, P-Value = 0.014; Fig. S6c). Moreover, the proportion of methylated CpGs correlated with age at disease onset (*r* = 0.59, P-Value = 0.020; Fig. 4b). Of note, age at collection and age at onset were strongly correlated (*r* = 0.98, P-Value = 8.06E-11; Fig. S6d). Hereafter, we leveraged our previously collected promoter methylation levels [8] to explore the relationship between methylation of the expansion and methylation of the promoter. We found that the proportion of methylated CpGs was in fact moderately positively correlated with promoter methylation (*r* = 0.41, P-Value = 0.033; Fig. S6e). In individuals with promoter hypermethylation, we found a trend toward higher expansion methylation levels (P-Value = 0.15; Fig. S6f), with a median proportion of methylated CpGs of 47% (22 to 66%) vs. 31% (13 to 62%).

#### Longitudinal and familial analyses

For 6 of the individuals, we obtained longitudinal specimens corresponding to 13 unique samples. We determined that for 4/6 of the individuals, the proportion of methylated CpGs was stable over time (< 10% change), while 2 individuals had more variable patterns (Fig. 4c), following the repeat length pattern (*see **blood expansion length and variability** section*). Furthermore, we assessed the amount of methylation between generations for 4 families corresponding to 7 different paternal transmissions of the expansion. Strikingly, all 7 of these transmissions demonstrated a lower proportion of methylated CpGs ($\:\ge\:$10% change) in the offspring compared to the father (Fig. 4d).

#### Cerebellum comparison

Our previous long-read sequencing study included 28 cerebellar samples [28]. We re-analyzed data from this study to obtain methylation levels (Fig. S7a-b). For the methylation analysis, we acquired 2,265 reads for the wild-type allele and 239 reads for the expanded allele. When comparing methylation levels between blood and cerebellum, we noticed that, for the wild-type allele, the median proportion of methylated CpGs was significantly higher in blood than in the cerebellum (P-Value = 3.01E-10; Fig. S7c). Similarly, for the expanded allele, a higher median proportion of methylated CpGs was seen in blood compared to the cerebellum (P-Value = 4.43E-09; Fig. S7c). This aligns with our observation that, for the three individuals included in both studies, the highest methylation levels were observed in blood, both for the wild-type and expanded alleles (Fig. S7d). Interestingly, we also noticed that for the expanded allele the amount of variation in the methylation levels, as measured by the range (maximum - minimum) within an individual was higher in the blood compared to the cerebellum (P-Value = 5.11E-05; Fig. S7e), but not for the wild-type allele (P-Value = 0.395; Fig. S7e).

### Repeat length

#### Blood expansion length and variability

**Fig. 5:**
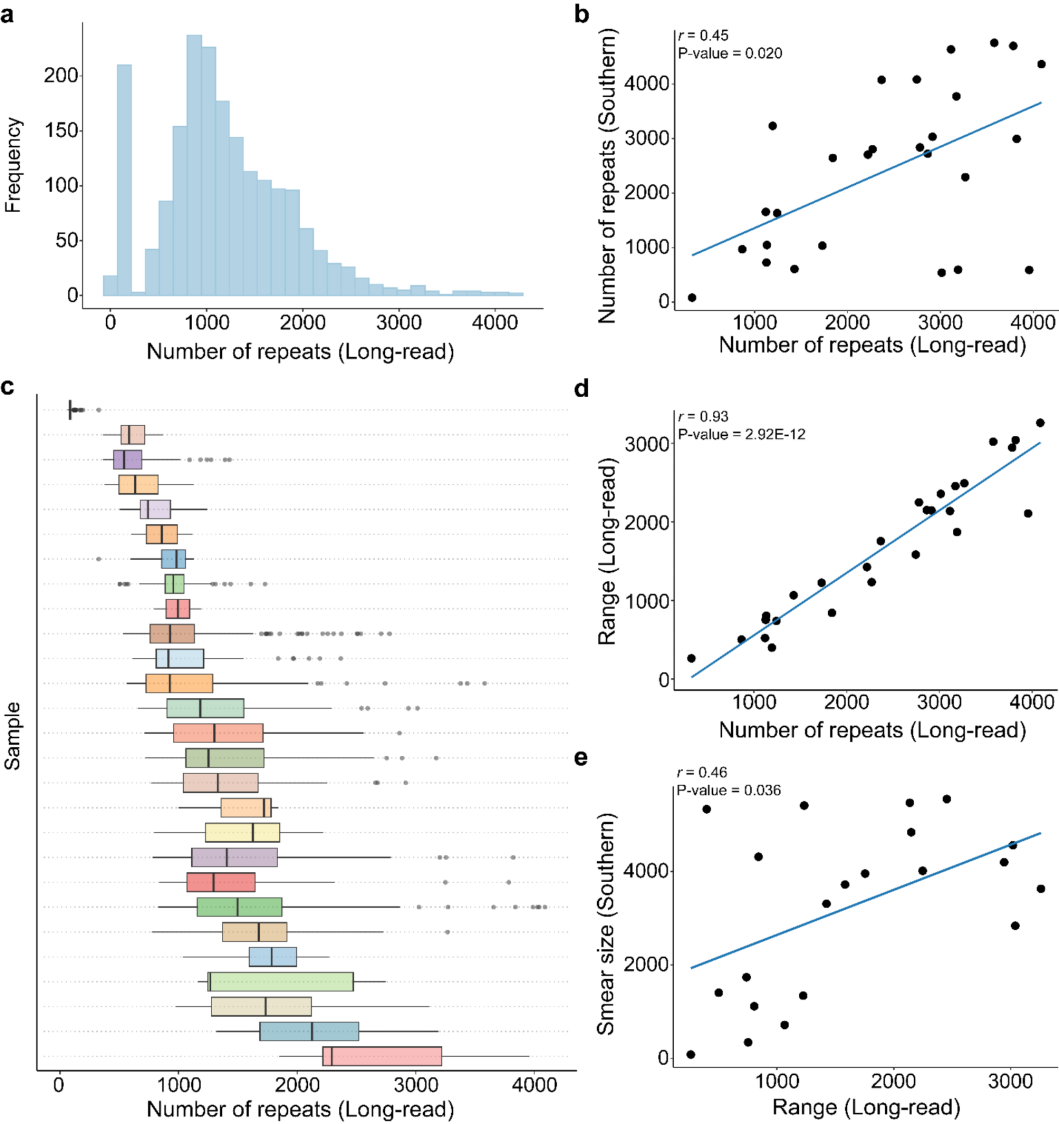
Repeat length analysis. (**a**) Histogram representing the number of repeats detected across all reads for every individual (*n* = 27) for the expanded allele. (**b**) Scatterplot displaying the number of repeats detected using long-read sequencing (maximum) and the number of repeats detected using Southern blotting. Each dot represents one individual (*n* = 27). A significant correlation was detected between the two estimates (*r* = 0.45, P-Value = 0.020). (**c**) Boxplot displaying the number of repeats for each read per individual (*n* = 27). Boxes represent the interquartile range (IQR; 25th − 75th percentile), lines represent the median. (**d**) Scatterplot displaying the range (maximum - minimm) of the number of repeats and the number of repeats detected using long-read sequencing (maximum). Each dot represents one individual (*n* = 27). A significant correlation was detected between the two estimates (*r* = 0.93, P-Value = 2.92E-12). The solid blue line represents a linear regression line. (**e**) Scatterplot displaying the range (maximum - minimum) of the number of repeats and the smear size detected in Southern blotting. A significant correlation was detected between the two estimates (*r* = 0.45, P-Value = 0.036). Each dot represents one individual (*n* = 27). The solid blue line represents a linear regression line. A Spearman’s rank correlation was used for each correlation analysis

After thoroughly analyzing the methylation level of the expanded *C9orf72* repeat, we then focused on the length of the expansion. We determined the length of both the wild-type and expanded alleles by counting the number of repeats between the first and last occurrence of a GGGGCC for all reads spanning the entire region. Importantly, the number of repeats detected for the wild-type allele using long-read sequencing was 100% concordant with fluorescent PCR measures (Fig. S8a). The expanded allele contained notable variability in terms of the number of repeats within a given individual. Overall, the distribution of read lengths appeared to be right tailed (Fig. 5a). In general, the concordance between Southern blotting length estimates and the maximum read length detected using long-read sequencing appeared to be better than using the median (Fig. 5b, Fig. S8b-c). Therefore, we completed our primary length analysis using the maximum estimate of the number of repeats for each individual. Across all subjects, the median number of maximum repeats detected was 2,746 (327 to 4,088; Fig. 5a, Table S3). We compared repeat lengths between Southern blotting estimates and long-read sequencing and found a significant positive correlation (*r* = 0.45, P-Value = 0.020; Fig. 5b). The measurements from long-read sequencing appeared to be similar in size to Southern blotting estimates (median = 2,746 long-read, median = 2,705 Southern blot), when using the maximum estimate (P-Value = 0.59; Fig. S7d).

As mentioned above, because we did notice a large amount of variation in the number of repeats within individuals (Fig. S9a-d), we assessed the variability of expansion length by measuring the range of the number of repeats for each individual. We determined that the median range of the number of repeats for each individual was 1424.5 (263.0 to 3260.5; Fig. 5c, Table S3). Notably, the range significantly increased as length measurements increased (*r* = 0.93, P-Value = 2.92E-12; Fig. 5d). We also determined the variability in the Southern blot data that we previously collected by analyzing the size of the smear for each individual. These measurements were available for 21/27 individuals. Interestingly, the range measurements from the long-read data were significantly positively correlated with these smear estimates (*r* = 0.46, P-Value = 0.036; Fig. 5e).

#### Blood associations

We completed additional associations with repeat length and clinical features and determined that, similar to the methylation analysis, the length of the expansion is positively correlated with age at collection (*r* = 0.62, P-Value = 6.32E-04; Fig. S10a) and seems smaller in pre-symptomatic individuals compared to symptomatic individuals (P-Value = 0.007; Fig. S10b). However, when we performed a multivariable linear regression analysis adjusting for age at collection, the difference between groups (presymptomatic vs. symptomatic) did not remain significant (P-Value = 0.57), suggesting that the significant difference in expansion length between pre-symptomatic and symptomatic individuals might be driven by their age difference. In the relatively small subset of patients with ALS (*n* = 15), we similarly identified a trend with age at collection (*r* = 0.39, P-Value = 0.15; Fig. S10c) and age at onset (*r* = 0.39, P-Value = 0.15; Fig. S10d).

#### Longitudinal and familial analyses

**Fig. 6:**
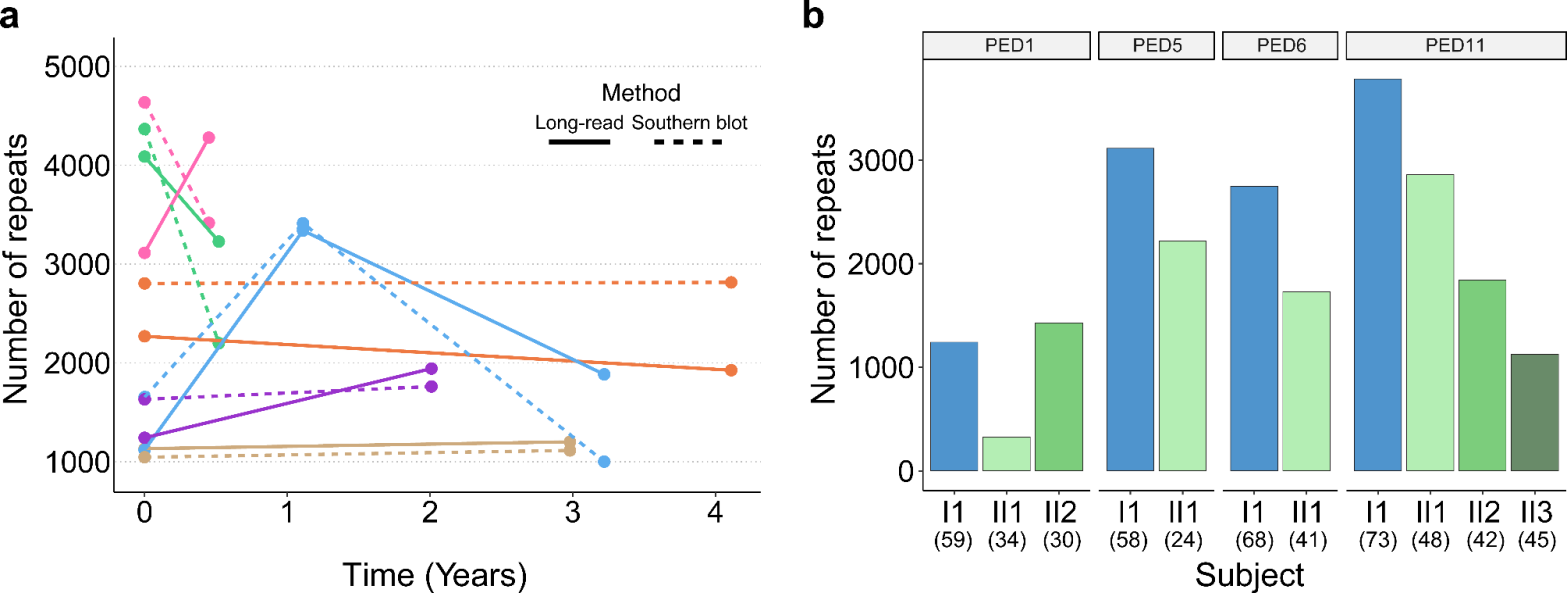
Repeat length longitudinal and familial analyses. (**a**) Dotplot showing the number of repeats detected using long-read sequencing (solid line) and Southern blotting (dashed line) over time. Longitudinal measurements were obtained for 6 individuals. Each dot represents the number of repeats detected (maximum) at a unique time point and lines connect a given individual. Individuals are assigned unique colors. (**b**) Barplot(s) showing the number of repeats per individual (maximum) in 4 pedigrees corresponding to 7 unique transmissions. Each pedigree was shown to display a paternally inherited contraction in our previous study. Paternal parents are presented as blue bars and offspring are presented in shades of green. Pedigree numbers are presented above each barplot and match our Southern blotting study. Numbers in parentheses represent age at collection for each individual

We then looked at the longitudinal data collected for the 6 individuals with multiple time points. Some individuals had stable expansions, while others fluctuated over time (Fig. 6a). Overall, the patterns of expansion lengths that we observed in the long-read sequencing data over time was generally well reflected in the Southern blotting estimates (5/6 subjects; Fig. 6a). Furthermore, we completed analyses within multiple families known to harbor the repeat expansion, focusing on the paternally inherited contractions we reported previously [8]. We detected contractions in 6/7 transmissions (Fig. 6b).

#### Cerebellum comparison

We then went back to our previously published cerebellar study to calculate the maximum repeat length [28]. We compared the length of the expansions detected in blood to those in the cerebellum and found that the median number of repeats in the blood was 2,746 vs. 1,932 in the cerebellum (Fig. S11a), which did not reach statistical significance in these modestly sized cohorts (P-Value = 0.11; Fig. S11b). A similar trend was observed when specifically focusing on the 3 overlapping subjects between regions (2,746 vs. 2,042; Fig. S11c). Like with methylation, we compared the variability of the number of repeats in the blood and cerebellum by measuring the range (maximum - minimum) for each sample. We detected significantly higher variability in the blood compared to the cerebellum (P-Value = 0.038; Fig. S11d).

### Sequence purity

#### Blood overview

**Fig. 7:**
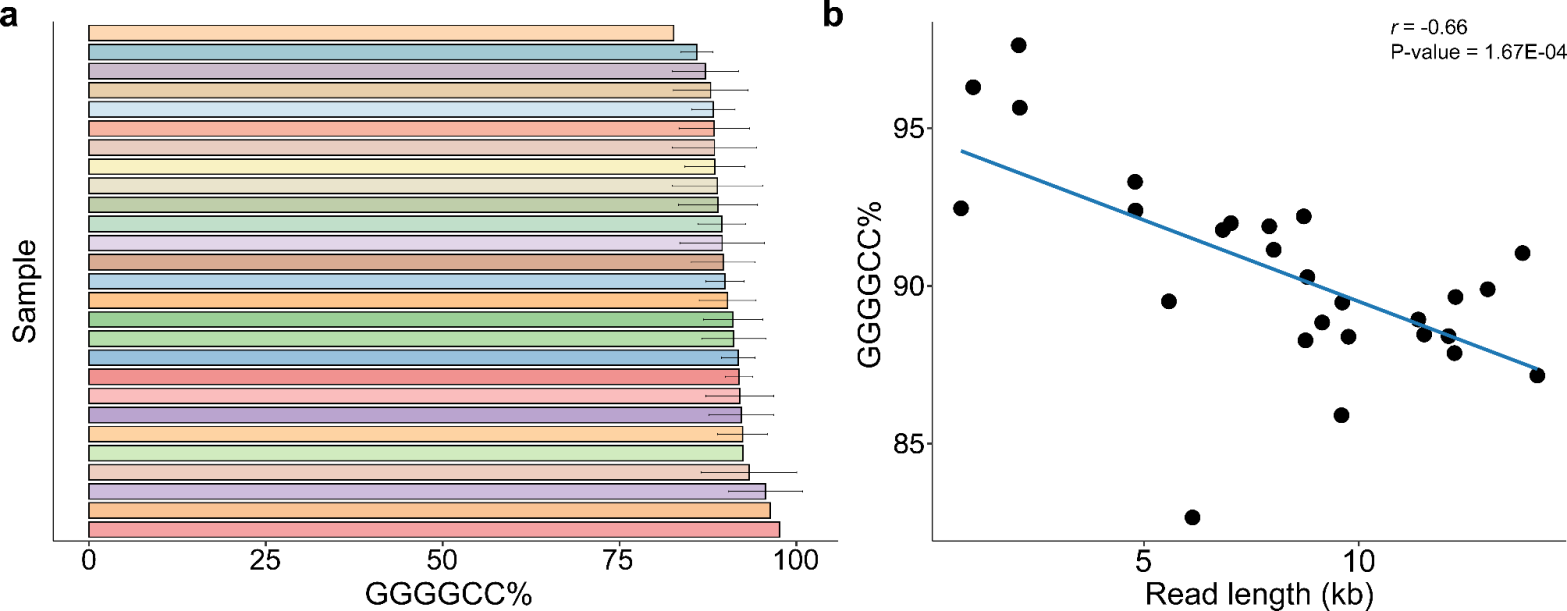
Sequence purity. (**a**) Barplot displaying the median percentage of the expansion composed of the GGGGCC motif per individual. Error bars represent the interquartile range (IQR; 25th − 75th percentile). Each individual has a unique color (*n* = 27). (**b**) Scatterplot displaying the percentage of the expansion composed of the GGGGCC motif for all reads and the read length (*n* = 27). Significantly higher purity was detected for shorter reads (*r* = -0.66, P-Value = 1.67E-04). Each dot represents one individual. The solid blue line represents a linear regression line. A Spearman’s rank correlation was used for this analysis

In addition to assessing the length of the expansion, we measured the purity of the sequence, using more stringent criteria when generating ccs reads (*see **methods*). We calculated the median GC% and GGGGCC% across all reads for each individual. We determined that the median GC% for the expanded allele was 100% (99.95–100%; Fig. S12a, Table S4) and 89.90% (82.66–97.63%) was composed of the GGGGCC repeat motif (Fig. 7a, Table S4). Unlike methylation and length, no associations with age or group were present. While longer reads did appear to be less pure than shorter reads (*r* = -0.66, P-Value = 1.67E-04; Fig. 7b), no correlation was present between methylation levels and sequence purity (*r* = -0.09, P-Value = 0.65; Fig. S12b). Looking at the longitudinal data, sequence purity appeared to be similar across time points in 5/6 subjects and stable between generations in all familial transmissions we assessed (Fig. S12c-d). Visualization of repeat purity did not reveal any notable patterns (Fig. S12e-f).

#### Cerebellum comparison

Again, we revisited our previously published cerebellar study to compare the sequence purity between regions [28]. When using the median, *C9orf72* expansions in the blood were less pure than in the cerebellum (P-Value = 8.22E-08), where the median GGGGCC% was 89.9% in the blood and 95.9% in the cerebellum. (Fig. S13a-b). The 3 overlapping subjects displayed a similar trend, with the median GGGGCC% in the blood (88.41%) lower than the cerebellum (96.05%; Fig. S13c). Notably, when analyzing the range (maximum - minimum) of the GGGGCC%, the opposite trend was revealed, where in blood the variability was significantly higher than in the cerebellum (P-Value = 3.92E-04; Fig. S13d).

## Discussion

In the current study, for the first time, we have quantitatively evaluated the methylation levels of the *C9orf72* expanded repeat. While methylation of the *C9orf72* promoter has been widely characterized [8, 43–46], limited studies have evaluated methylation of the repeat expansion itself [47]. One study was able to determine that the expansion was methylated using a PCR-based method but was unable to quantify the amount of methylation [47]. Here, we clearly demonstrated that the expanded allele contains CpG sites that can be methylated (Fig. 2a-b). Of note, this allele appears to have extensive variation in the amount of methylation detected between individuals and regions. We determined that in blood, the expanded allele appears to be more highly methylated than the wild-type allele across nearly all individuals investigated (Fig. 2c-e). In Fragile X syndrome, another disease caused by a long, GC-rich repeat expansion, researchers have determined that expansions only become methylated after 200 repeats are present, which is then considered to be a full mutation [48, 49]. Perhaps a similar phenomenon can be observed in the current study, where the individual with the smallest repeat expansion had the lowest median methylation score and a relatively low proportion of methylated CpGs (Fig. 3a, S5a-c). Though, our data suggests that methylation levels are positively correlated with repeat length, further studies that include more individuals with smaller repeats are necessary to determine if there is a point at which methylation of the expansion is present, like in Fragile X syndrome.

We found that methylation levels positively correlate with age at collection (Fig. 4a) and age at disease onset (Fig. 4b). Even though we also observed a difference in methylation levels between pre-symptomatic and symptomatic individuals, it seemed to be driven by the difference in age at collection. Our modest collection of longitudinal and familial specimens allowed us to begin exploring the expansion over time and between generations (Figs. 4c and 6a). We found that the amount of methylation over time was stable in some individuals, and dynamic in others. In the familial data, where we previously observed contractions in the repeat length, we also observed lower amounts of methylation in offspring compared to the parents (Figs. 4d and 6b). One important consideration to make when interpreting this observation is that the ages of the offspring tended to be lower than the ages of the parents. Therefore, given the identified associations with age at collection, more detailed studies of a larger number of subjects, including many longitudinal and familial specimens, will be crucial in fully untangling these findings.

We then re-analyzed our previously published cohort of cerebellar long-read sequencing data [28] to determine the amount of methylation in this region. There appeared to be less methylation of the expansion in the cerebellum than in blood (Fig. S7c). We cannot exclude the possibility that based on our Southern blot data this might be driven by the fact that *C9orf72* expansions tend to be smaller in the cerebellum than in blood [23]. One could speculate that this may be due to differences in cellular composition and architecture between tissues [59, 60], with more terminally differentiated cells (i.e., neurons) in the cerebellum, possibly resulting in smaller, more stable expansions, which is in line with our observation of less variability within a sample (Fig. S7e). It would, therefore, be of interest to perform targeted long-read sequencing studies on specific cell populations, which may aid to elucidate these findings.

For our repeat length analysis, importantly, we observed that the long-read sequencing determined length reflected our previous Southern blotting observations, demonstrating the power of this technology to accurately size repeat expansions [8]. Our current study provided insight into observed variation in repeat length within an individual (Fig. 5c-e). Specifically, there was a significant positive correlation between the size of smears observed using Southern blotting and the range of repeat length detected by long-read sequencing, suggesting the smear patterns represent real variation in repeat length. One possibility is that repeat length differs between unique cell types, as it does between brain regions, blood, and other tissues [24, 54–56, 61–63]. As mentioned above, future studies of individual cell types derived from human tissue will be important to understand this phenomenon.

Our correlation analyses of repeat length revealed similar trends to our observations with methylation levels. Using repeat length, we identified correlations with age at collection (Fig. S10a) and a difference between pre-symptomatic and symptomatic individuals (Fig. S10b), although the latter appeared to be driven by age at collection. Here, we confirmed our previous observations using Southern blotting that expansions in some individuals may be dynamic over time. Additionally, we confirmed observations of paternally inherited contractions in our familial specimens.

Finally, we evaluated the purity of the sequence within the repeat expansion. We detected very few interruptions within the repeat expansion (Fig. 7a, S12a). Those that we did find were primarily single nucleotide changes (e.g., single nucleotide deletions) to the GGGGCC motif. Unlike other repeat expansions such as myotonic dystrophy type 1 [64], there does not appear to be a clear pattern to these interruptions even between reads of the same individual (Fig. S12e-f). Perhaps this is due to the length variation between reads within an individual, therefore, alignment of the interruptions remains a challenge. However, as the repeat motif serves as a template for RAN translation, sequence impurities could potentially modify DPR production. Notably, longer reads tended to be less pure, but no clinical associations were identified. More work should be done to understand the relevance of these small alterations, especially in primary affected regions.

We acknowledge that our study has some limitations. While we already observed multiple relevant associations, given our modest sample size, we may have missed others. For example, we observed many similarities between repeat length and methylation levels in the blood, as the two measures were positively correlated (*r* = 0.67, P-Value = 1.18E-04). Thus, it is difficult to determine the driving factor of the observed associations. It is likely that larger studies are needed to more carefully tease apart this relationship. In addition, we let our previous Southern blot study guide us in selecting samples of interest for our present targeted long-read sequencing study, which may have resulted in selection bias. Because most of our subjects were either pre-symptomatic or had ALS, we were unable to evaluate differences in disease subtype (e.g., ALS vs. FTD). Future large-scale studies, therefore, should include well-balanced groups of subjects spanning the entire ALS-FTD-disease spectrum. It is likely that larger-scale longitudinal studies that include pheno-converters are needed to fully understand the dynamics of the expansion over time, with age, and between pre-symptomatic and symptomatic individuals. One technical limitation of the current study was that PacBio’s long-read sequencing method only permitted us to measure 5mC at CpGs, even though other DNA modifications at additional locations may exist. Additionally, symmetric methylation of both strands is assumed, therefore potential hemi-methylated sites were unable to be identified. Of course, using targeted approaches limited our ability to evaluate the methylation profile of these individuals at other locations in the genome. As both PacBio and other long-read sequencing technologies (e.g., ONT) rapidly advance, additional types of modifications, potentially at a genome-wide scale, will be evaluated [41]. Finally, while we believe studying the blood of clinical patients is important because it is easily accessible and gives us access to longitudinal measurements, one additional limitation of our study is that we did not sequence DNA extracted from primary affected regions in the ALS-FTD disease spectrum (e.g., motor cortex, spinal cord, frontal cortex, and temporal cortex). Future studies should be performed for those regions to similarly characterize the methylation, length and purity of the *C9orf72* repeat expansion.

## Conclusions

Overall, long-read sequencing allowed us to thoroughly characterize the *C9orf72* repeat expansion. Our study provides the first quantitative evaluation of *C9orf72* repeat expansion methylation at a resolution not achievable with previously used methods and suggests that it may be associated with relevant clinical features (e.g., age at collection, age at onset). While we acknowledge that much larger studies are needed to fully understand the clinical impacts of this work, it may be important to consider methylation of this repeat expansion as a potential disease modifier, pending further evaluation. Of note, the expansion appeared to be more highly methylated in the blood compared to the cerebellum. Therefore, future studies of primary affected brain regions and even specific cell types will be crucial in resolving the relevance of our findings.

## Electronic Supplementary Material

Below is the link to the electronic supplementary material.

Supplementary Material 1

Supplementary Material 2

## Data Availability

Additional information is available upon reasonable request.

## Abbreviations

5mC: 5-methylcytosine
ALS: Amyotrophic lateral sclerosis
C9orf72: C9orf72-SMCR8 complex subunit
ccs: Circular consensus sequence
DPR: Dipeptide repeat
FTD: Frontotemporal dementia
iPSC: Induced pluripotent stem cell
IQR: Interquartile range
No-Amp: No-amplification
ONT: Oxford Nanopore Technologies
PacBio: Pacific Biosciences
PED: Pedigree
RAN: Repeat association non-AUG
SMRT: Single-molecule real-time
