# Supplementary material for: Targeted long-read sequencing to quantify methylation of the C9orf72 repeat expansion
Source: Mol Neurodegener. 2024 Dec 21;19:99. doi: 10.1186/s13024-024-00790-0 (PMC11663317; doi:10.1186/s13024-024-00790-0)
Supplement: Supplementary file 2 — Supplementary Material 2 [file 13024_2024_790_MOESM2_ESM.docx]

**Targeted long-read sequencing to quantify methylation of the *C9orf72* repeat expansion**

Evan Udine^1,2^, NiCole A. Finch^1^, Mariely DeJesus-Hernandez^1^, Jazmyne L. Jackson^3^, Matthew C. Baker^1^, Siva Arumugam Saravanaperumal^4^, Eric Wieben^4^, Mark T.W. Ebbert^5^, Jaimin Shah^6^, Leonard Petrucelli^1,2^, Rosa Rademakers^1,7,8^, Björn Oskarsson^6^, Marka van Blitterswijk^1,2^

**Affiliations:** ^1^ Mayo Clinic Department of Neuroscience, Jacksonville, FL, ^2^ Neuroscience Ph.D. Program, Mayo Clinic Graduate School of Biomedical Sciences, Mayo Clinic, ^3^ Fels Cancer Institute for Personalized Medicine, Temple University, Lewis Katz School of Medicine, Philadelphia, PA, ^4^ Mayo Clinic Genome Analysis Core, Rochester, MN, ^5^ University of Kentucky Sanders-Brown Center on Aging, Department of Neuroscience, Lexington, KY, ^6^ Mayo Clinic Department of Neurology, Jacksonville, FL, ^7^ VIB Center for Molecular Neurology, Antwerp, Belgium, ^8^ University of Antwerp Department of Biomedical Science, Antwerp, Belgium

**Corresponding Author:** Marka van Blitterswijk, M.D., Ph.D., Department of Neuroscience, Mayo Clinic, 4500 San Pablo Rd S, Jacksonville, FL 32224, USA. Telephone: +1 904-953-2226. Email: [VanBlitterswijk.Marka@mayo.edu](mailto:VanBlitterswijk.Marka@mayo.edu)

**Table Legends.**

**Table S1.** Blood wild-type allele methylation summary statistics. Summary statistics are provided for the analysis of wild-type allele methylation, including the read length, median methylation score per read (MedianMethylationScore), and the proportion of methylated CpGs to total CpGs (MedianPropMethylatedCpGs) as indicated in the variable column. The per subject mean, standard deviation (SD), median, interquartile range (IQR), 90^th^, and 100^th^ percentile (P90-P100) are presented in individual columns.

**Table S2.** Blood expanded allele methylation summary statistics. Summary statistics are provided for the analysis of expanded allele methylation, including the read length, median methylation score per read (MedianMethylationScore), and the proportion of methylated CpGs to total CpGs (MedianPropMethylatedCpGs) as indicated in the variable column. The per subject mean, standard deviation (SD), median, interquartile range (IQR), 90^th^, and 100^th^ percentile (P90-P100) are presented in individual columns.

**Table S3.** Blood expansion length summary statistics. Summary statistics are provided for the analysis of expansion length, including total read length, expansion length, and the number of repeats, as indicated in the variable column. The per subject mean, standard deviation (SD), median, interquartile range (IQR), 90^th^, and 100^th^ percentile (P90-P100) are presented in individual columns.

**Table S4.** Blood expansion purity summary statistics. Summary statistics are provided for the analysis of sequence purity, including GC% and GGGGCC%, as indicated in the variable column. The per subject mean, standard deviation (SD), median, interquartile range (IQR), 90^th^, and 100^th^ percentile (P90-P100) are presented in individual columns.


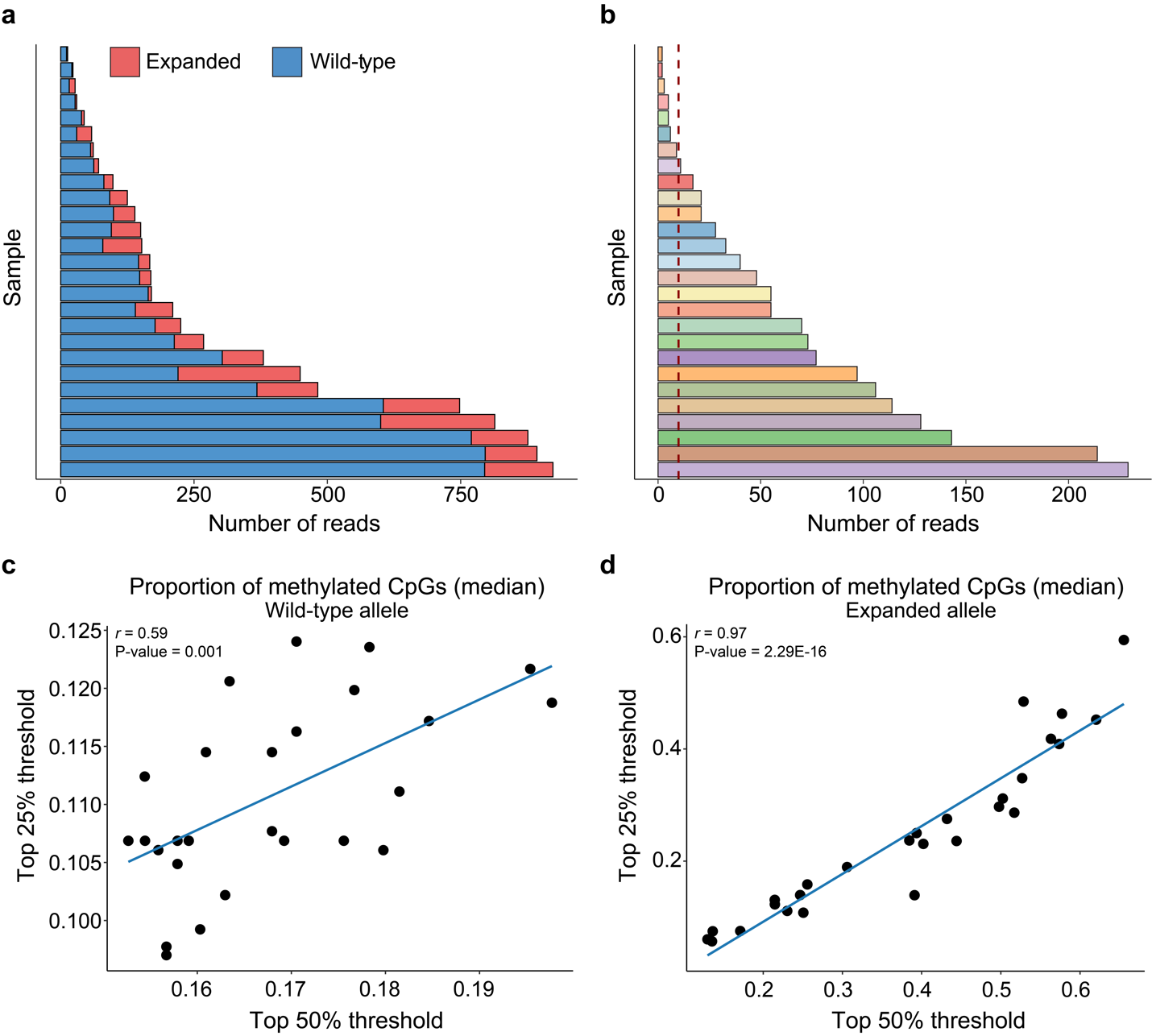
**Figure S1.**

**Figure S1.** Number of reads overview. (**a**) Barplot showing the number of reads per individual (n = 27) for both the wild-type allele (blue) and expanded allele (red) for the *C9orf72* target region. (**b**) Barplot displaying the number of reads covering the expanded allele per individual. Each individual has a unique color (n = 27). The dashed red line represents 10 reads. (**c-d**) Scatterplot showing the median proportion of methylated CpGs per read for each individual. The x-axis represents the proportion when considering scores greater than or equal to 128 (top 50%) to be methylated, while the y-axis displays the proportion when considering scores greater than or equal to 192 (top 25%) to be methylated. Significant positive correlations were detected for both the (**c**) wild-type (*r* = 0.59, P-Value = 0.001) and (**d**) expanded alleles (*r* = 0.97, P-Value = 2.29E-16). The solid blue line(s) represents a linear regression line. Spearman’s rank correlations were used for these analyses.


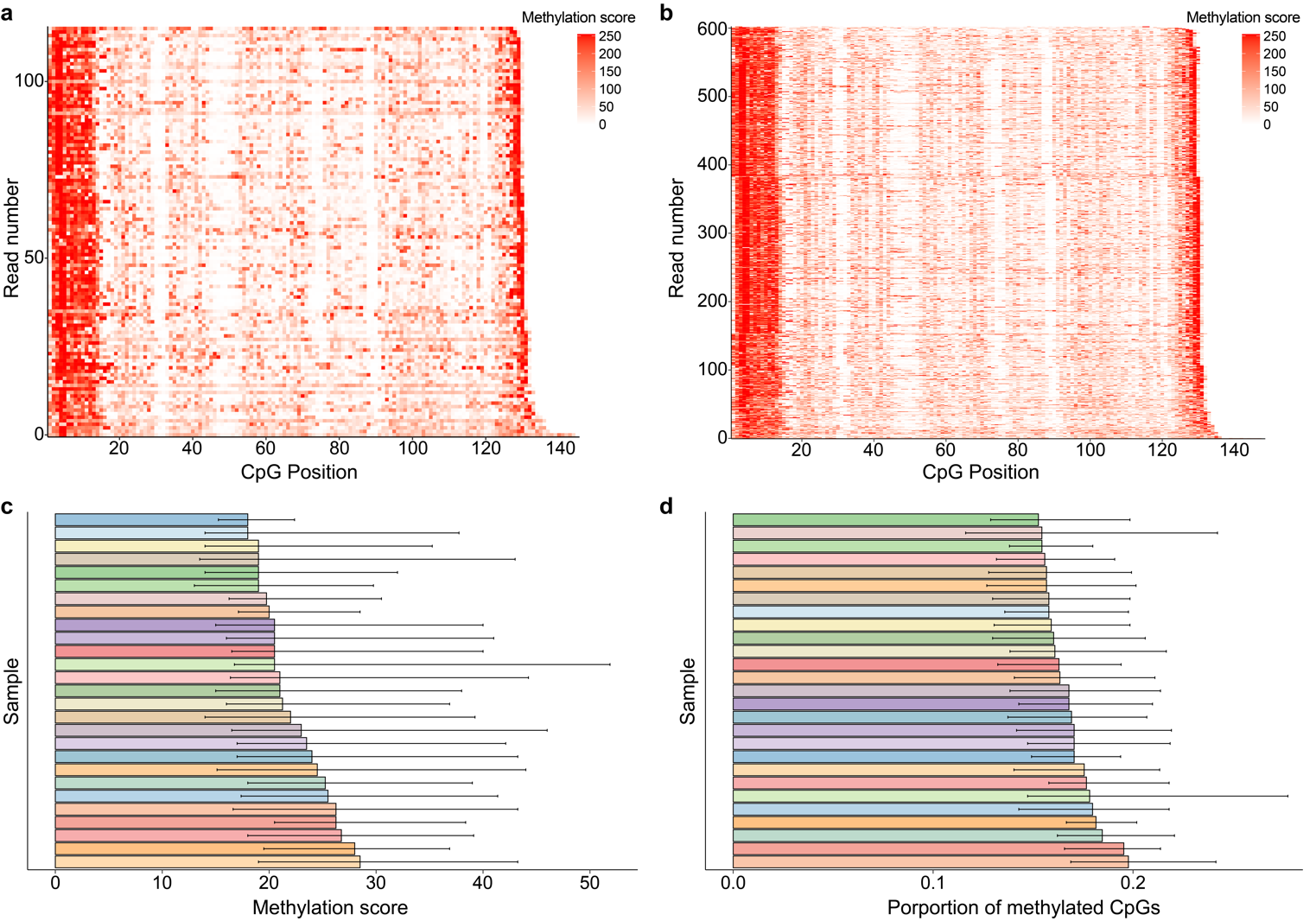
**Figure S2.**

**Figure S2.** Wild-type allele methylation. (**a-b**) Waterfall-like plots for the wild-type allele (with flanking region) for two representative individuals. The x-axis represents the position of each CpG within a read and the y-axis displays all reads sorted by number of CpG sites. Low methylation scores are presented in white and higher scores in red. (**c-d**) Barplots showing (**c**) the median methylation score per read and (**d**) the median proportion of methylated CpGs per read (n = 27) per individual. Each individual has a unique color. Error bars represent the interquartile range (IQR; 25^th^ - 75^th^ percentile).

**
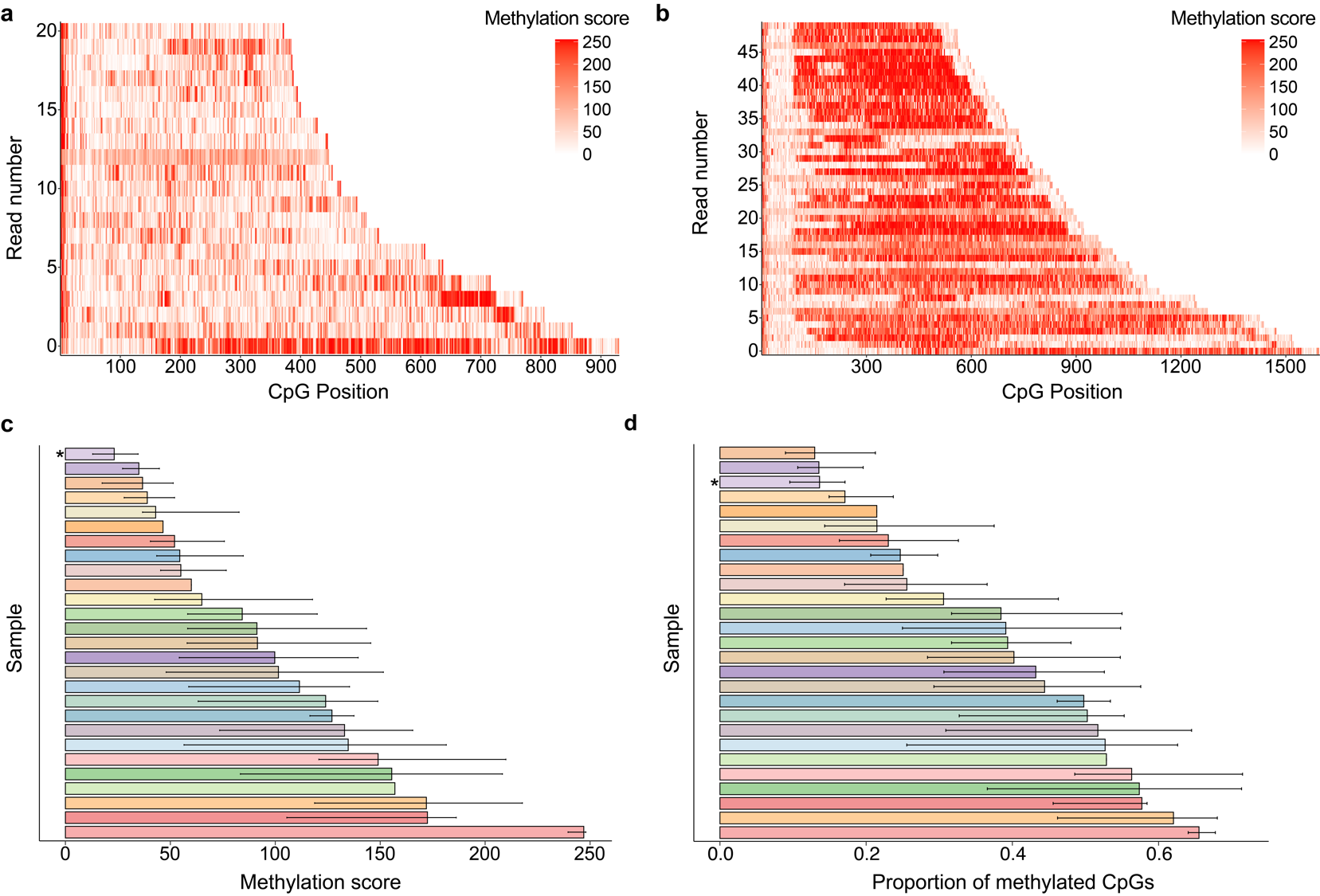
Figure S3.**

**Figure S3.** Expanded allele methylation. (**a-b**) Waterfall-like plots for the expanded allele (with flanking region) for two representative individuals. The x-axis represents the position of each CpG within a read and the y-axis displays all reads sorted by number of CpG sites. Low methylation scores are presented in white and higher scores in red. (**c-d**) Barplots showing (**c**) the median methylation score per read and (**d**) the median proportion of methylated CpGs per read (n = 27). Error bars represent the interquartile range (IQR; 25^th^ - 75^th^ percentile). The asterisk indicates the sample with the smallest repeat expansion.

**
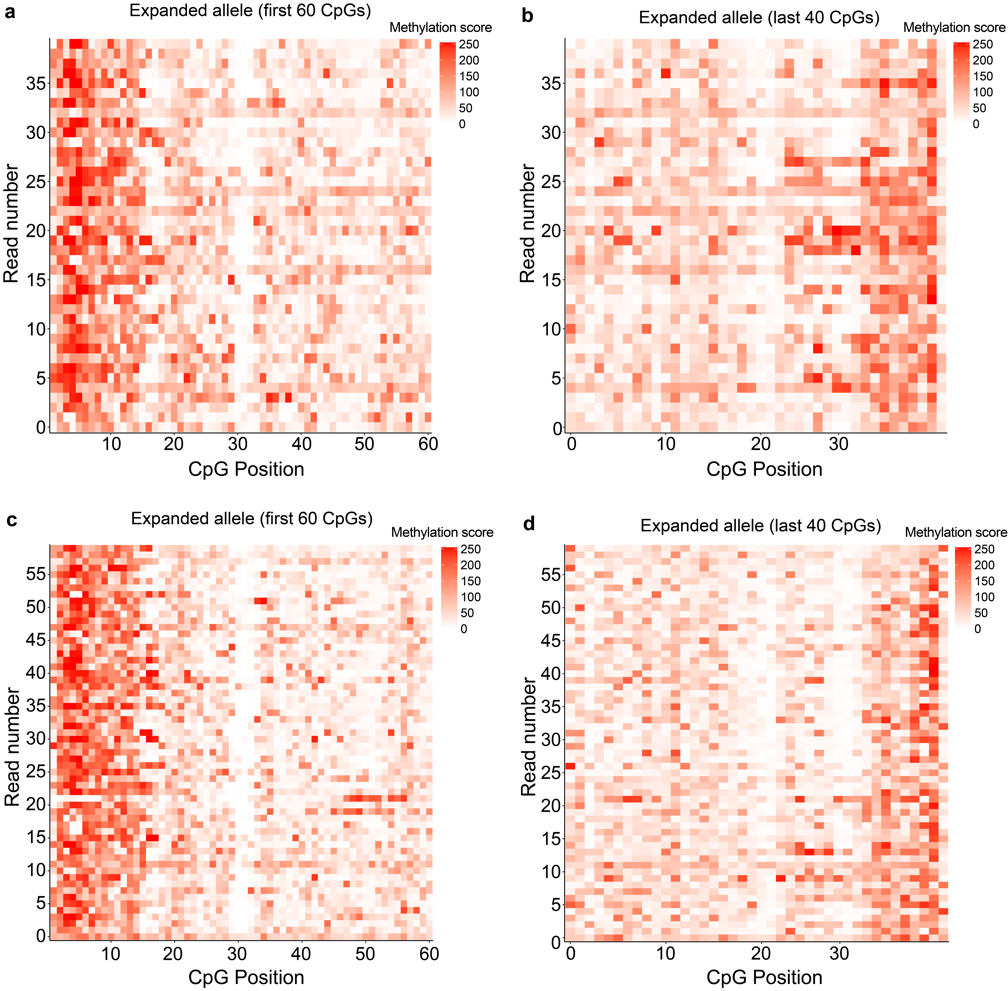
Figure S4.**

**Figure S4.** Methylation patterns at the ends of the *C9orf72* repeat expansion. (**a-d**) Waterfall-like plots for the (**a,c**) first 60 CpGs and (**b,d**) last 40 CpGs of each read for the expanded allele from (**a-b**) the individual included in Figure 2b and (**c-d**) the individual presented in Figure 3b. The x-axis represents the position of each CpG within a read and the y-axis displays all reads sorted by number of CpG sites Low methylation scores are presented in white and higher scores in red.

**Figure S
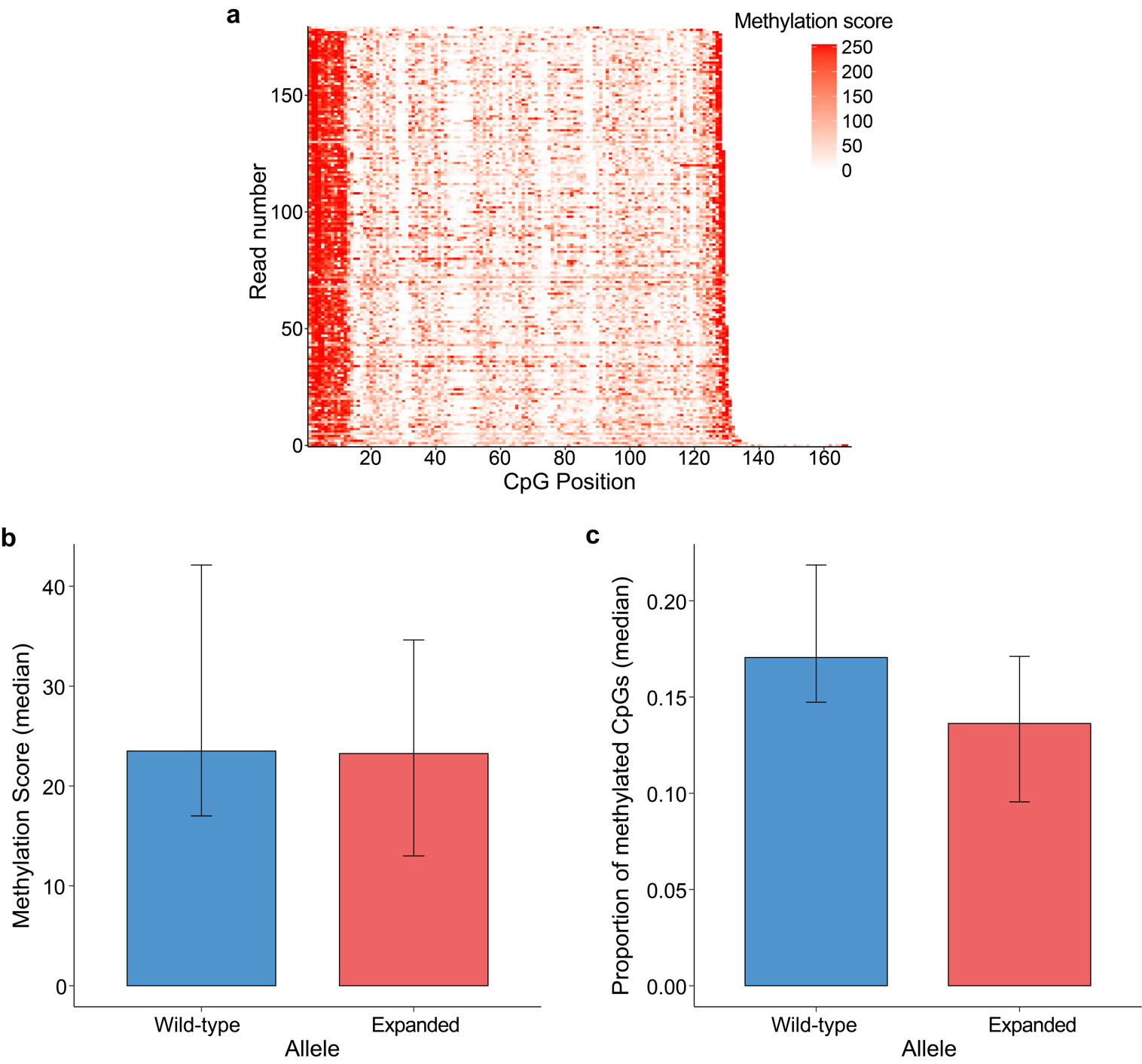
5.**

**Figure S5.** Methylation of one individual with a small expansion and low level of methylation. (**a**) Waterfall-like plot for the wild-type allele (with flanking region). The x-axis represents the position of each CpG within a read and the y-axis displays all reads sorted by number of CpG sites. Low methylation scores are presented in white and higher scores in red. (**b-c**) Barplots showing (**b**) the median methylation score per read (**c**) and the median proportion of methylated CpGs per read for each allele. Error bars represent the interquartile range (IQR; 25^th^ - 75^th^ percentile).

**
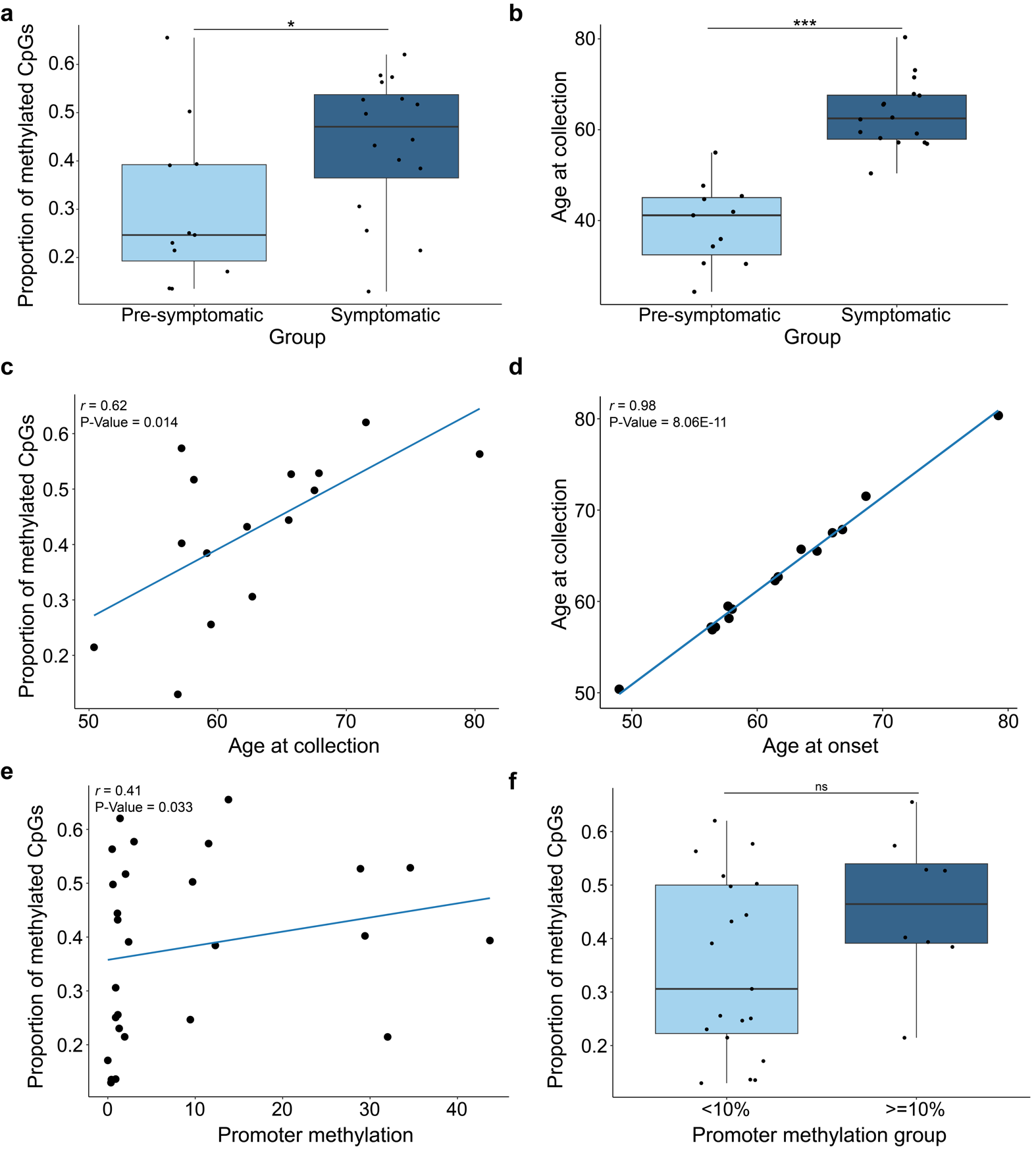
Figure S6.**

**Figure S6.** Expanded allele methylation associations. (**a**) Boxplot displaying the median proportion of methylated CpGs per read for each individual for the expanded allele for pre-symptomatic and symptomatic individuals. Boxes represent the interquartile range (IQR; 25^th^ - 75^th^ percentile), lines represent the median, and each dot corresponds to one individual. Significantly higher methylation was detected in symptomatic individuals (n = 16) compared to pre-symptomatic individuals (n = 11; P-Value = 0.041). (**b**) Boxplot displaying the age at collection for each individual comparing pre-symptomatic and symptomatic individuals. Boxes represent the interquartile range (IQR; 25^th^ - 75^th^ percentile), lines represent the median, and each dot corresponds to one individual. Symptomatic individuals (n = 16) had a significantly higher age at collection compared to pre-symptomatic individuals (n = 11; P-Value = 1.97E-05). (**c**) Scatterplot showing the median proportion of methylated CpGs per read for patients with ALS (n = 15) for the expanded allele and the age at collection. A significant positive correlation was detected (*r* = 0.62, P-Value = 0.014). The solid blue line represents a linear regression line. (**d**) Scatterplot showing the age at collection and age at onset for patients with ALS (n = 15). A significant positive correlation was detected (*r* = 0.98, P-Value = 8.06E-11). The solid blue line represents a linear regression line. (**e**) Scatterplot showing the median proportion of methylated CpGs per read for each individual for the expanded allele and promoter methylation. A significant positive correlation was detected (*r* = 0.41, P-Value = 0.033). The solid blue line represents a linear regression line. (**f**) Boxplot displaying the median proportion of methylated CpGs per read for each individual (hypomethylation n = 19, hypermethylation n = 8) for the expanded allele split by promoter methylation group. Boxes represent the interquartile range (IQR; 25^th^ - 75^th^ percentile), lines represent the median, and each dot corresponds to one individual. A trend for higher methylation in individuals with promoter hypermethylation was present (P-Value = 0.15). A Wilcoxon rank-sum test was used for group comparisons and a Spearman’s rank correlation was used for correlation analyses. *P-Value < 0.05, ***P-Value < 0.001; ns = P-Value > 0.05


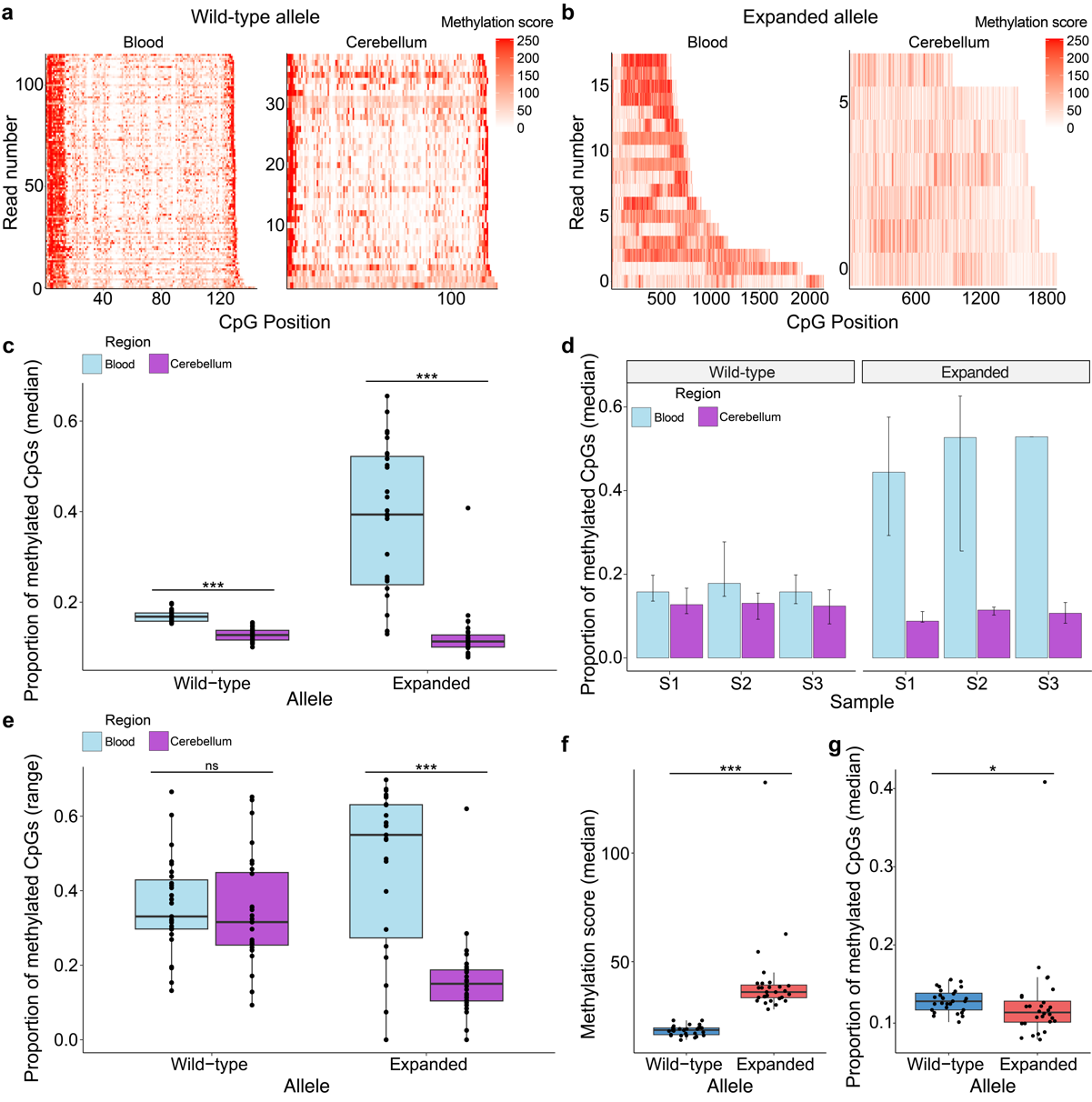
**Figure S7.**

**Figure S7.** Comparison of methylation in the blood and brain**.** (**a-b**) Waterfall-like plots for the (**a**) wild-type and (**b**) expanded alleles (with flanking region) for one representative individual in each region. The x-axis represents the position of each CpG within a read and the y-axis displays all reads sorted by number of CpG sites. Low methylation scores are presented in white and higher scores in red. Blood methylation plots are displayed on the left of each panel and cerebellum methylation plots are displayed on the right for each panel. (**c**) Boxplot displaying the median proportion of methylated CpGs per read for every individual for each allele (blood n = 27; cerebellum n = 28). Boxes represent the interquartile range (IQR; 25^th^ - 75^th^ percentile), lines represent the median, and each dot corresponds to one individual. We detected higher methylation for the wild-type (P-Value = 3.01E-10) and expanded (P-Value = 4.43E-09) allele(s) in the blood. A Wilcoxon rank-sum test was used for this comparison. (**d**) Barplot displaying the median proportion of methylated CpGs per read for each allele for individuals with both blood and cerebellar tissue (n = 3). Bars extend to the median and error bars represent the interquartile range (IQR; 25^th^ - 75^th^ percentile). (**e**) Boxplot displaying the range (maximum - minimum) of the proportion of methylated CpGs per read for every individual for each allele (blood n = 27; cerebellum n = 28). We detected more variability of methylation for the expanded allele in the blood (P-Value = 5.11E-05), but no significant difference for the wild-type allele (P-Value = 0.395). A Wilcoxon rank-sum test was used for this comparison. (**f-g**) Boxplot(s) displaying (**f**) the median methylation score and (**g**) median proportion of methylated CpGs per read for each individual (n = 28) for each allele. Higher methylation was detected for the expanded allele using the methylation score (P-Value = 3.98E-06) while a higher proportion of methylated CpGs was detected for the wild-type allele (P-Value = 0.04). It should be noted, however, that the observed difference between alleles regarding the proportion of methylated CpGs might be due to the fact that the total number of CpGs is higher for the expanded allele. A paired Wilcoxon rank-sum test was used for each of these comparisons. Boxes represent the interquartile range (IQR; 25^th^ - 75^th^ percentile), lines represent the median, and each dot corresponds to one individual. ***P-Value < 0.001, *P-Value < 0.05, ns = P-Value > 0.05

**
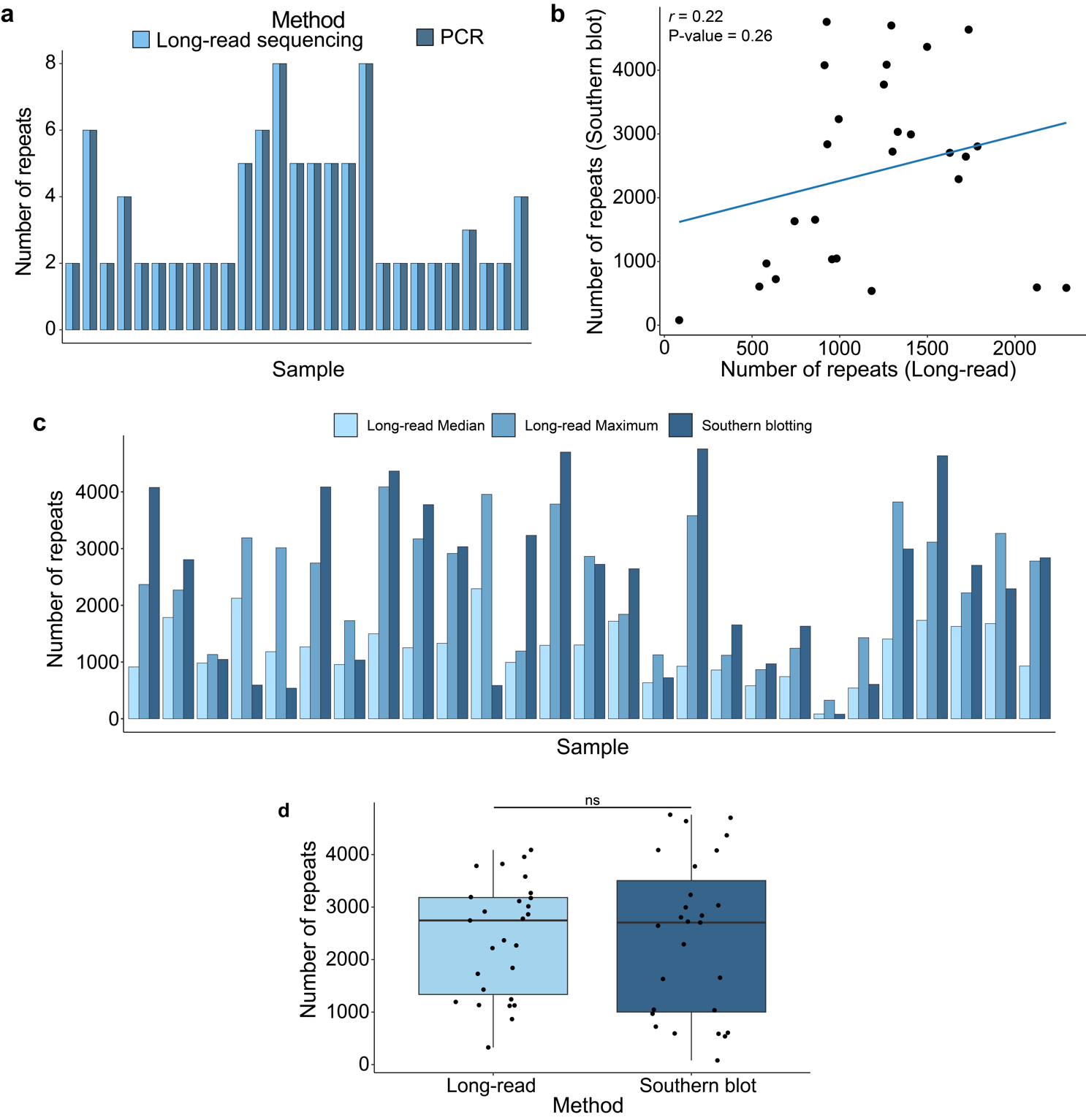
Figure S8.**

**Figure S8.** Long-read repeat length estimates. (**a**) Barplot showing the number of repeats detected per individual (n = 27) using long-read sequencing and fluorescent PCR. (**b**) Scatterplot displaying the number of repeats detected using long-read sequencing (median estimate) and the number of repeats detected using Southern blotting. Each dot represents one individual (n = 27). A significant correlation was not detected between the two estimates (*r* = 0.22, P-Value = 0.26). The solid blue line represents a linear regression line. A Spearman’s rank correlation was used for this analysis. (**c**) Barplot showing the number of repeats detected per individual (n = 27) using the median number of repeats detected using long-read sequencing estimates (light blue), the maximum number of repeats using long-read sequencing (medium blue), and Southern blotting (dark blue). (**d**) Boxplots comparing the number of repeats detected using long-read sequencing (maximum) and the number of repeats detected using Southern blotting. Boxes represent the interquartile range (IQR; 25^th^ - 75^th^ percentile), lines represent the median, and each dot represents one individual (n = 27). No significant difference was found (P-Value = 0.59). A paired Wilcoxon rank-sum test was used for this comparison. ns = P-Value > 0.05

**
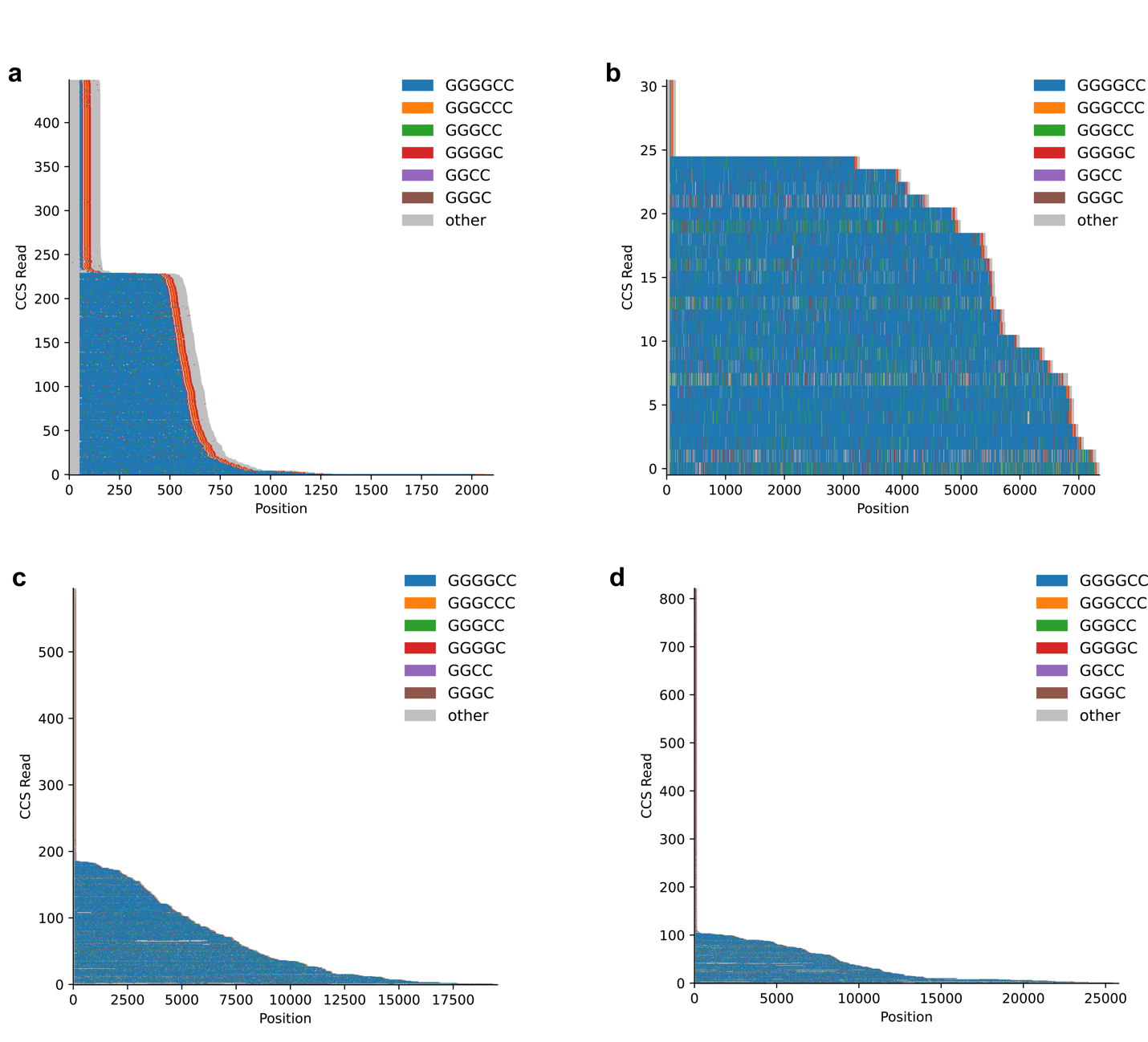
Figure S9.**

**Figure S9.** *C9orf72* length distributions. (**a-d**) Waterfall plots for four representative individuals, showing the position of each nucleotide for the wild-type and expanded alleles, with the flanking region on the x-axis and each unique read stacked on top of each other on the y-axis. Reads are sorted in descending order by read length. The primary repeat motif (GGGGCC) is colored blue, with alternative motifs in additional colors.

**
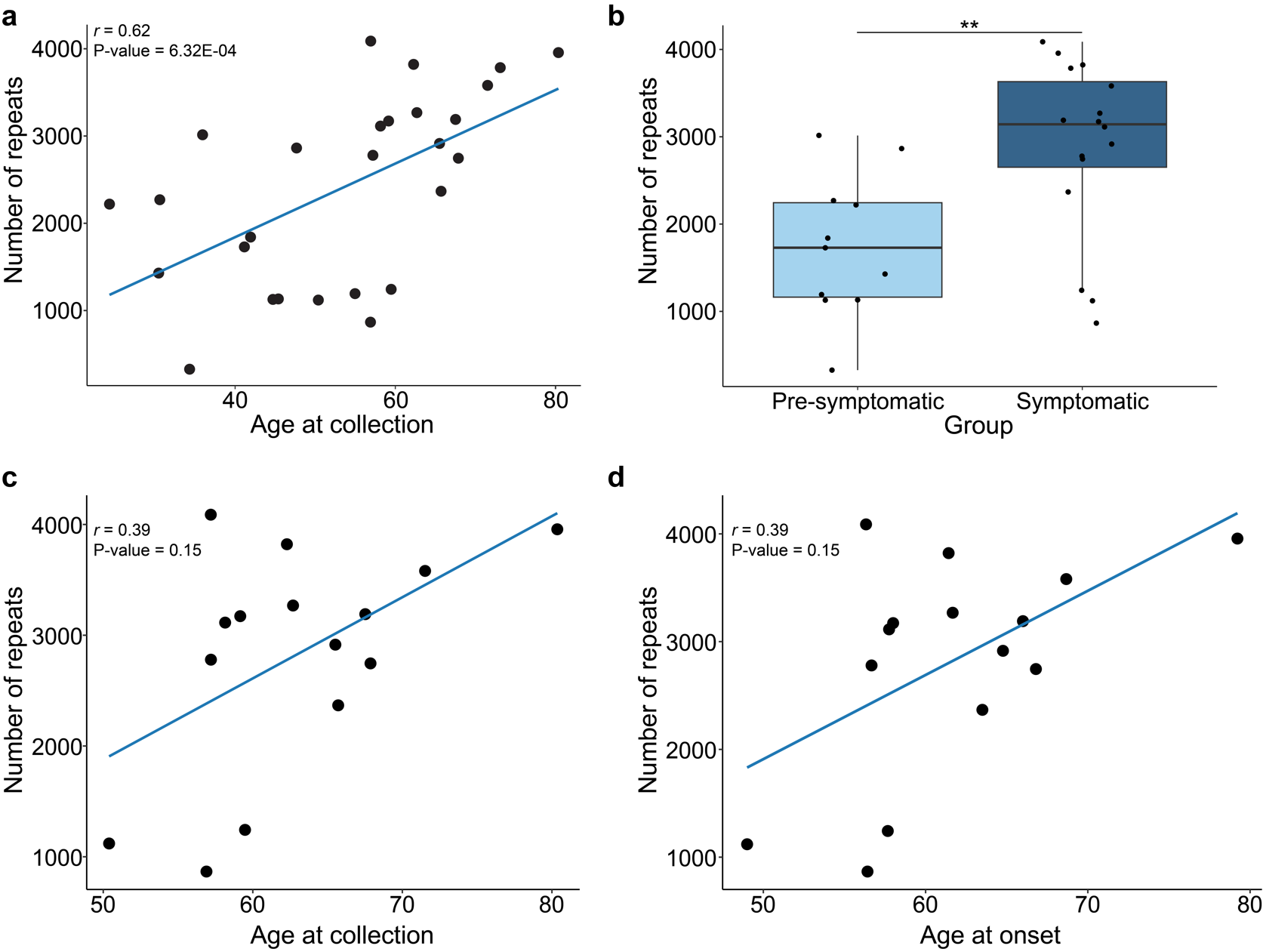
Figure S10.**

**Figure S10.** Repeat length associations. (**a**) Scatterplot displaying the age at collection and the number of repeats (maximum) detected using long-read sequencing. Each dot represents one individual (n = 27). A significant correlation was detected between the two estimates (r = 0.62, P-Value = 6.32E-04). The solid blue line represents a linear regression line. (**b**) Boxplot comparing the number of repeats (maximum) between pre-symptomatic (n = 11) and symptomatic individuals (n = 16; P-Value = 0.007). Boxes represent the interquartile range (IQR; 25^th^ - 75^th^ percentile), lines represent the median, and each dot represents one individual. A Wilcoxon rank-sum test was used for this comparison. (**c**) Scatterplot showing the number of repeats (maximum) for patients with ALS (n = 15) for the expanded allele and the age at collection. A significant positive correlation was not detected (r = 0.39, P-Value = 0.15). The solid blue line represents a linear regression line. (**d**) Scatterplot showing the number of repeats (maximum) for patients with ALS (n = 15) for the expanded allele and age at onset. A significant positive correlation was not detected with age at onset (r = 0.39, P-Value = 0.15). The solid blue line represents a linear regression line. A Spearman’s rank correlation was completed for each correlation analysis. **P-Value < 0.01

**
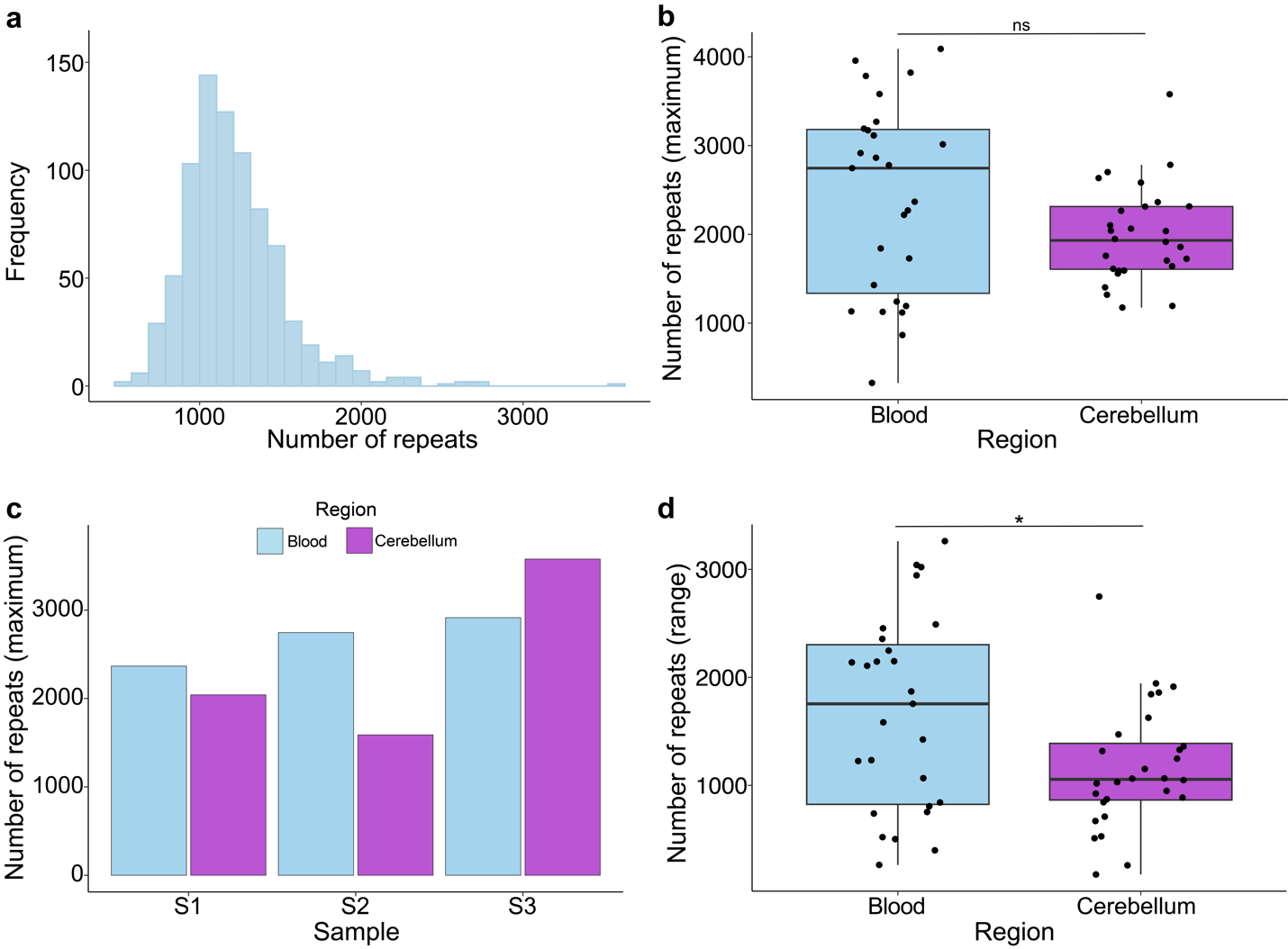
Figure S11.**

**Figure S11.** Cerebellum length analysis. (**a**) Histogram representing the number of repeats detected across all reads for every individual (n = 28) for the expanded allele. The expanded allele number of repeats ranges from approximately 1,176 - 3,578 repeats. (**b**) Boxplots displaying the number of repeats for all individuals (blood n = 27, cerebellum n = 28). Boxes represent the interquartile range (IQR; 25^th^ - 75^th^ percentile), lines represent the median, and each dot corresponds to one individual. No significant differences were detected between regions (P-Value = 0.11). (**c**) Barplot displaying the maximum number of repeats for individuals with both blood and cerebellar tissue (n = 3). Bars extend to the median and error bars represent the interquartile range (IQR; 25^th^ - 75^th^ percentile). (**d**) Boxplots displaying the range (maximum - minimum) of the number of repeats for all individuals (blood n = 27, cerebellum n = 28). Boxes represent the interquartile range (IQR; 25^th^ - 75^th^ percentile), lines represent the median, and each dot corresponds to one individual. A significant higher range was detected in the blood compared to the cerebellum (P-Value = 0.038). A Wilcoxon rank-sum test was used for each comparison. *P-Value < 0.05, ns = P-Value > 0.05

**
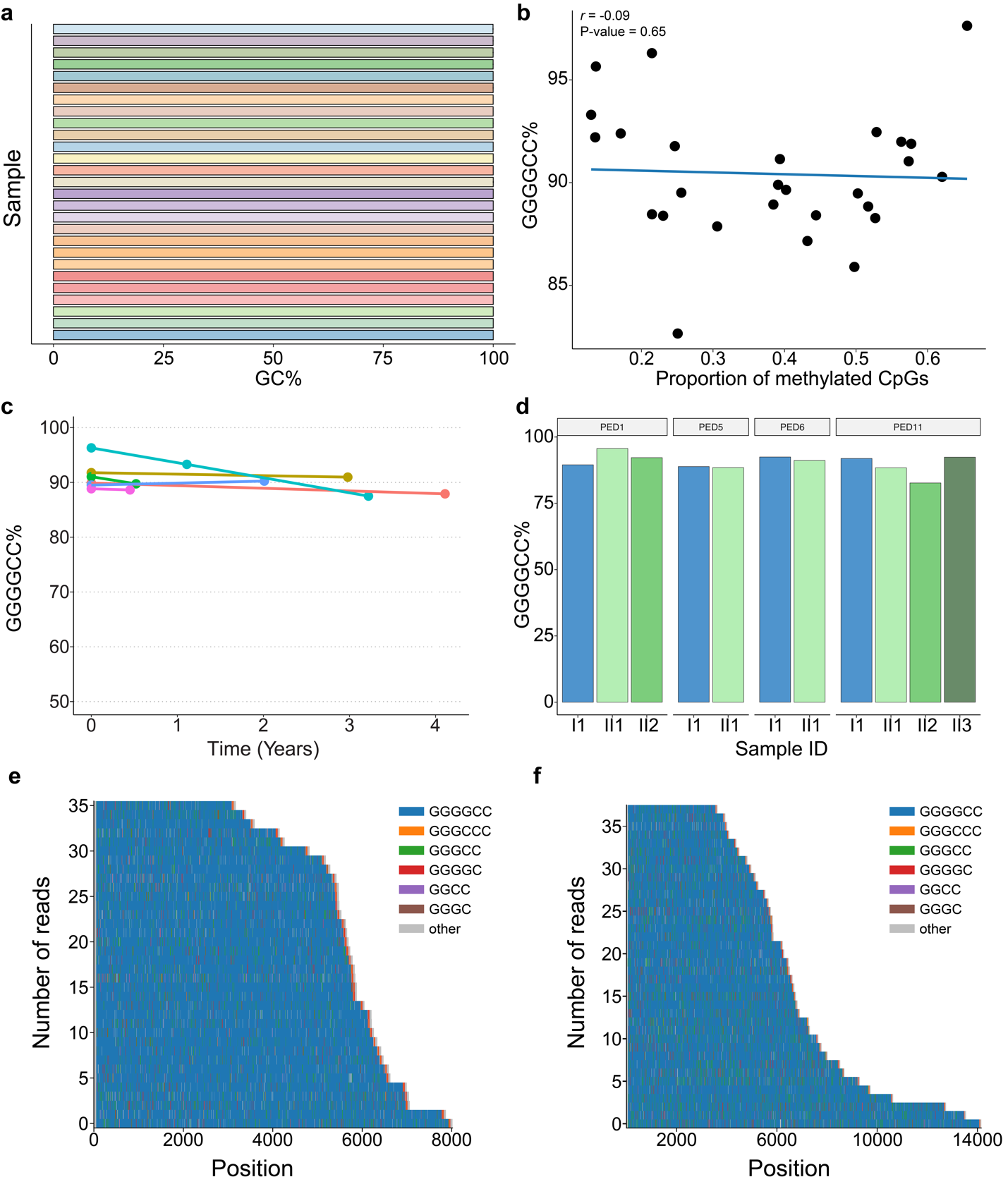
Figure S12.**

**Figure S12.** Blood sequence purity analysis. (**a**) Barplot displaying the median GC percentage of the expansion. Error bars represent the interquartile range (IQR; 25^th^ - 75^th^ percentile). Each individual has a unique color (n = 27). (**b**) Scatterplot displaying the percentage of the expansion composed of the GGGGCC motif for all reads and the GGGGCC% (n = 27). No significant correlation was detected (*r* = -0.09, P-Value = 0.65; Spearman’s rank correlation). The solid blue line represents a linear regression line. (**c**) Dotplot showing the median GGGGCC% per read for each individual for the expanded allele over time measured in years. Longitudinal measurements were obtained for 6 subjects. Each dot represents a unique time point and lines connect the points within a given individual. Each individual is assigned a unique color. (**d**) Barplot(s) showing the median GGGGCC% per read for each individual across 4 different pedigrees corresponding to 7 unique transmissions. Each pedigree was shown to display a paternally inherited contraction in our previous Southern blotting study. Paternal parents are presented as blue bars and offspring are presented in various shades of green. (**e-f**) Waterfall plots for two individuals, showing the position of each nucleotide for the expanded allele and flanking region on the x-axis for each unique read (y-axis). Reads are sorted in descending order by read length. The primary repeat motif (GGGGCC) is colored blue, with alternative motifs in additional colors. No clear pattern of interruptions is observed.


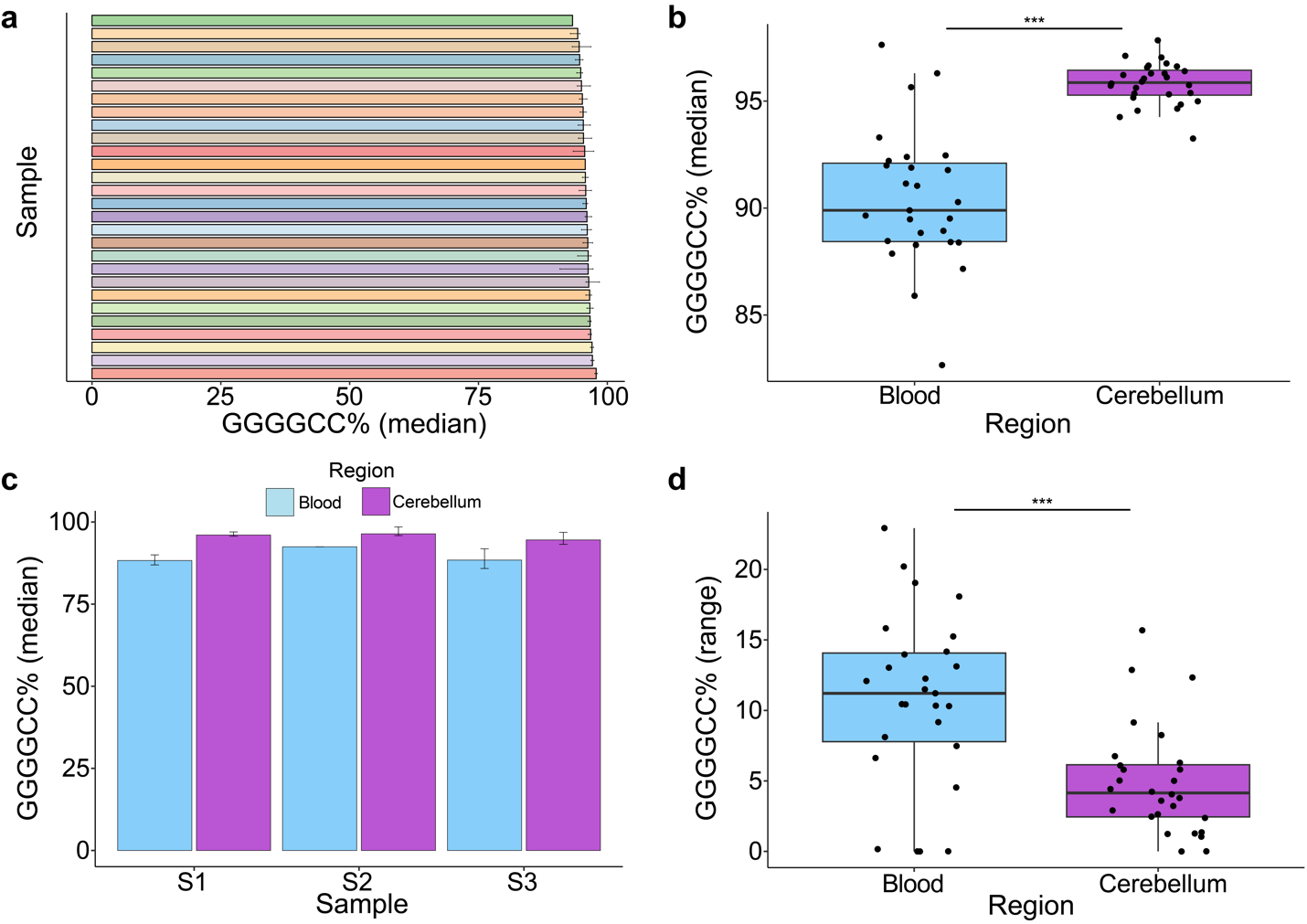
**Figure S13.**

**Figure S13**. Cerebellum sequence purity. (**a**) Barplot displaying the median percentage of the expansion composed of the GGGGCC motif per individual. Lines represent the interquartile range (IQR; 25^th^ - 75^th^ percentile). Each individual has a unique color (n = 28). (**b**) Boxplot displaying the median repeat purity (GGGGCC%) for all individuals (blood n = 27, cerebellum n = 28). Boxes represent the interquartile range (IQR; 25^th^ - 75^th^ percentile), lines represent the median, and each dot corresponds to one individual. A significant difference was detected between regions when including all individuals (P-Value = 8.22E-08). (**c**) Barplot displaying the median repeat purity (GGGGCC%) for individuals with both blood and cerebellar tissue (n = 3). Bars extend to the median and error bars represent the interquartile range (IQR; 25^th^ - 75^th^ percentile). (**d**) Boxplot displaying the range (maximum - minimum) of repeat purity (GGGGCC%) for all individuals (blood n = 27, cerebellum n = 28). Boxes represent the interquartile range (IQR; 25^th^ - 75^th^ percentile), lines represent the median, and each dot corresponds to one individual. A significant difference was detected between regions (P-Value = 3.92E-04). A Wilcoxon rank-sum test was used for each comparison. ***P-Value  < 0.001
